# Enhanced insights into the genetic architecture of 3D cranial vault shape using pleiotropy-informed GWAS

**DOI:** 10.1038/s42003-025-07875-6

**Published:** 2025-03-15

**Authors:** Seppe Goovaerts, Sahin Naqvi, Hanne Hoskens, Noah Herrick, Meng Yuan, Mark D. Shriver, John R. Shaffer, Susan Walsh, Seth M. Weinberg, Joanna Wysocka, Peter Claes

**Affiliations:** 1 Department of Human Genetics, KU Leuven, Leuven, Belgium; 2 Medical Imaging Research Center, University Hospitals Leuven, Leuven, Belgium; 3 Department of Chemical and Systems Biology, Stanford University School of Medicine, Stanford, CA USA; 4 Departments of Genetics and Biology, Stanford University School of Medicine, Stanford, CA USA; 5 Division of Gastroenterology, Hepatology, and Nutrition, Boston Children’s Hospital, Boston, MA USA; 6 Department of Pediatrics, Harvard Medical School, Boston, MA USA; 7 Department of Electrical Engineering, ESAT/PSI, KU Leuven, Leuven, Belgium; 8 Department of Cell Biology & Anatomy, Cumming School of Medicine, Alberta Children’s Hospital Research, Institute, University of Calgary, Calgary, AB Canada; 9 Department of Biology, Indiana University Indianapolis, Indianapolis, IN USA; 10 Center for Craniofacial and Dental Genetics, Department of Oral and Craniofacial Sciences, University of Pittsburgh, Pittsburgh, PA USA; 11 Department of Anthropology, Pennsylvania State University, State College, PA USA; 12 Department of Human Genetics, University of Pittsburgh, Pittsburgh, PA USA; 13 Department of Anthropology, University of Pittsburgh, Pittsburgh, PA USA; 14 Department of Developmental Biology, Stanford University School of Medicine, Stanford, CA USA; 15 Howard Hughes Medical Institute, Stanford University School of Medicine, Stanford, CA USA; 16 Murdoch Children’s Research Institute, Melbourne, VIC Australia

**Keywords:** Genome-wide association studies, Development

## Abstract

Large-scale GWAS studies have uncovered hundreds of genomic loci linked to facial and brain shape variation, but only tens associated with cranial vault shape, a largely overlooked aspect of the craniofacial complex. Surrounding the neocortex, the cranial vault plays a central role during craniofacial development and understanding its genetics are pivotal for understanding craniofacial conditions. Experimental biology and prior genetic studies have generated a wealth of knowledge that presents opportunities to aid further genetic discovery efforts. Here, we use the conditional FDR method to leverage GWAS data of facial shape, brain shape, and bone mineral density to enhance SNP discovery for cranial vault shape. This approach identified 120 independent genomic loci at 1% FDR, nearly tripling the number discovered through unconditioned analysis and implicating crucial craniofacial transcription factors and signaling pathways. These results significantly advance our genetic understanding of cranial vault shape and craniofacial development more broadly.

## Abstract (web summary)

Leveraging prior genetic associations with brain shape, facial shape, and bone mineral density enhances the discovery of genes and pathways underlying human cranial vault shape variation.

## Introduction

The intricacies of human development and evolution are conspicuously evident in the head, where multiple tissue types and organs co-develop and co-evolve in unison to ensure structural and functional coherence^1,2^. For example, the increased brain size in humans, relative to other primates, was accommodated by the co-evolution of craniofacial skeletal features, such as a domed cranium and increased basicranial flexion^2^. Epigenomic divergence related to key craniofacial genes underlies these features and genomic variation related to gene regulation is an important source of craniofacial differences within and between human populations today^3–7^. Knowledge of specific genes and variants and how they affect the different components of the head is key to understanding its development as a complex system.

Formation of the human head starts early in development when the rostral end of the neural tube forms the hindbrain, midbrain, and forebrain, the latter eventually developing into the cerebral hemispheres^8^. From the same neural tube region, cranial neural crest cells (CNCCs) delaminate and migrate ventrally^9,10^. Anterior-most CNCCs form the frontonasal skeleton, while posterior CNCCs populate the pharyngeal arches to form the bone and cartilage of the jaws^10^. The rate of growth of the early brain influences the positioning of facial structures^11^, while the flat bones of the neurocranium, derived from the paraxial mesoderm, are joined by flexible sutures that accommodate brain expansion^12^. Dysregulated coordination between the brain and craniofacial mesenchyme results in congenital malformations such as cleft lip and palate or craniosynostosis^11–14^. Throughout development, many genes have roles that span multiple structures of the human head, influencing both their development and integration. For example, by controlling neural tube development, *ZIC2* and *ZIC3* affect the common origins of the brain and skull^15,16^, while *SOX9* has independent roles in brain^17^ and facial^18^ development. Additionally, a plethora of key facial genes (e.g., *DLX5*, *RUNX2*, and *TWIST1*) and signaling pathways (e.g., BMP/TGF-β, FGF, and Wnt) also affect suture fusion and are implicated in craniosynostosis^18,19^. Together, these extensive genetic and morphological relationships are crucial to consider when studying the genetic basis of craniofacial variation as they present both opportunities and challenges for genetic discovery and interpretation.

Genome-wide association studies (GWAS) have been instrumental in identifying the genetic underpinnings of complex traits^20^, including highly heritable cranial vault dimensions^21^. While initial attempts relied on simple anthropometric traits^22–27^, a recent GWAS significantly advanced genomic discovery by extracting three-dimensional (3D) cranial vault shape from whole-head magnetic resonance (MR) images^6^. Current findings from GWAS implicate important signaling pathways to contribute to cranial vault shape variation (e.g., FGF, BMP/TGF-β, Wnt, Hedgehog) and affirm the key role of RUNX2, a master regulator of calvarial ossification^28–32^. We have also shown that many of the identified loci are shared between the face, brain, and cranial vault and are closely related to craniosynostosis, in line with current knowledge on developmental biology^6,33^. These findings suggest that further investigation into cranial vault shape genetics may offer valuable insights into head development as a system and the etiology of craniofacial conditions. However, the tens of loci identified for cranial vault shape remain far fewer than those identified for facial^7,34–43^ and brain^33,44–51^ phenotypes, revealing a critical gap in cranial vault genetics.

In this study, we aim to leverage prior biological and genetic information to enhance the discovery of genomic loci underlying cranial vault morphology. Given the extensive genomic overlap between brain, facial, and cranial vault morphology^6^, as well as evidence that genes associated with bone mineral density (BMD) control cranial suture ossification^52,53^, we demonstrate that single nucleotide polymorphisms (SNPs) associated with those traits have an increased likelihood of association with cranial vault shape. In an empirical Bayesian framework, this can be interpreted as evidence in favor of a positive association with cranial vault shape resulting in a posterior, or conditional false discovery rate (FDR) that is decreased with respect to the prior, or unconditioned FDR. We then apply these principles through means of the conditional FDR method^54–61^ to a recent cranial vault shape GWAS^6^ to leverage GWAS data on brain shape, facial shape, and BMD, thereby revealing novel associated genes and pathways.

## Results

### Cranial vault shape associated SNPs are enriched among SNPs associated with related traits

**Fig. 1:**
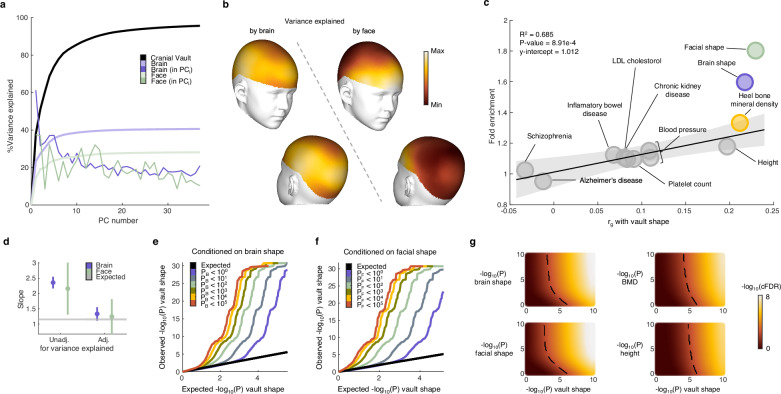
The genetic architectures of brain and facial shape are enriched for SNPs associated with cranial vault shape because of genomic and morphological correlations. **a** Cumulative cranial vault (black) shape variation explained by brain (purple) and facial (green) shape. Light green and purple show the total cranial vault shape variation explained by the face and brain, cumulatively over all cranial vault shape PCs. Dark green and purple show the variance explained in the i^th^ PC of the cranial vault. **b** Heatmap of variance explained by brain and facial shape per vertex on the cranial vault. The same color scheme applies to the whole panel. **c** The fold enrichment of SNPs with *P*_vault_ < 0.05 amongst SNPs with *P*_other_ < 0.05 versus all SNPs is plotted against genomic Spearman correlations with cranial vault shape. GWAS sample sizes are as follows: cranial vault shape (*n* = 6772), facial shape (*n* = 8246), brain shape (*n* = 19,644), heel bone mineral density (*n* = 426,824), height (*n* = 2,200,007), systolic blood pressure (*n* = 385,798), diastolic blood pressure (*n* = 385,801), chronic kidney disease (*n* = 64,164 cases + 561,055 controls), platelet count (*n* = 408,112), LDL cholesterol (*n* = 361,194), inflammatory bowel disease (*n* = 25,042 cases + 34,915 controls), Alzheimer's disease (*n* = 71,880 cases + 383,378 controls), schizophrenia (*n* = 36,989 cases + 113,075 controls). The trend line and 95% CI were estimated using iteratively reweighted least squares (*P* = 8.91e–4). **d** GWAS summary data from 63 hierarchical modules of facial shape and 285 hierarchical modules of brain shape were used to estimate and adjust the fold enrichment from (**c**) for the cranial vault shape variance explained by the brain or facial shape modules. Error bars represent the 95% CI. **e** Q-Q plot of *P* values from the cranial vault shape GWAS conditioned on the *P* values with brain shape and **f** facial shape. Each color in **e** and **f** corresponds to the set of SNPs from the cranial vault shape GWAS that satisfies the *P* value criterion in the auxiliary GWAS. **g** Conditional false discovery rate (cFDR) as a function of a SNP’s *P* value for cranial vault shape and for a conditioning trait. Dashed lines indicate the 1% cFDR boundary.

Using previously described pipelines^6,33,62^, we extracted the facial, cranial vault, and mid-cortical surface from the T1-weigthed MR-images of the Adolescent Brain Cognitive Development Study (ABCD Study)^63,64^. Based on the sets of morphological features extracted through principal component analysis (PCA), we estimated the proportion of cranial vault shape variation explained by the brain’s shape (Fig. 1a). This estimation was performed cumulatively across the principal components (PCs) of cranial vault shape. Brain shape explained 40.6% of the overall cranial vault shape variation and explained more variation in the first few PCs individually, i.e., up to 61.3% of the variation in the first PC. Furthermore, as shown in Fig. 1b, shape variation explained by the brain at a vertex-level was consistently high across the cranial vault. Together, these findings illustrate that the major modes of cranial vault shape variation, which define its overall dimensions, are reflected in the shape of the underlying cortical surface. In contrast, the 28.1% of cranial vault shape variation explained by facial shape was less focused on the first few PCs (Fig. 1a) and limited mostly to the forehead and regions in close proximity to the face (Fig. 1b).

Next, we investigated whether the genetic architectures of complex traits and diseases were enriched for SNPs associated with cranial vault shape. For this purpose, GWAS summary statistics were obtained from our recent cranial vault shape GWAS^6^ and from GWAS studies^7,33,65–73^ on other complex phenotypes conducted in independent cohorts. The fold enrichment of statistical association was defined as the proportion of SNPs with *P*_vault_ < 0.05 in the set of SNPs with *P*_other_ < 0.05 versus in the set of all SNPs. We observed a strong enrichment of statistical association with cranial vault shape amongst the SNPs associated with other craniofacial and skeletal traits, including brain shape, facial shape, BMD, and height (Fig. 1c). As these enrichments are directly related to the Bayesian principles of the conditional FDR method used later in this work, we aimed to investigate how they corresponded to genomic Spearman correlations^33^, another method for assessing genetic overlap that is applicable to multivariate GWAS. Unsurprisingly, genomic Spearman correlations were strongly correlated with cross-trait enrichments of association (R^2^ = 0.685; *P* = 8.91e–4; Fig. 1c). These results demonstrate that when a trait is genetically correlated with cranial vault shape, the information on a SNP’s positive association with that trait increases its empirical likelihood to be positively associated with cranial vault shape.

For brain and facial shape, the enrichments were substantially higher relative to their genomic correlations with cranial vault shape. To investigate this overlap further, we downloaded GWAS summary statistics for 63 hierarchical facial shape modules^7^ and 285 hierarchical brain shape modules^33^, ranging from the entire brain and face to more localized segments, such as the nose, and local regions of the cortical surface (Supplementary Fig. 1). These smaller segments of the brain and face varied in the strength of their genomic and morphological correlations with cranial vault shape, both of which had a positive effect on the enrichments. Moreover, we observed that in the presence of morphological correlations, the observed fold enrichments were inflated relative to what our regression model from Fig. 1c predicted based on the genomic correlation alone. Only after adjusting for the strength of the morphological correlation, did the genomic correlation predict fold enrichments in agreement with our earlier model (Fig. 1d). This illustrates that the high fold enrichments observed for the brain and face could be explained by their morphological correlations with the cranial vault and hence that morphological integration is a significant source of cross-trait associations. We also note that despite the variability in sample size in Fig. 1c, we obtained consistent estimates of the slope when using GWAS data with constant sample sizes (Fig. 1d), demonstrating robustness of the initial estimate.

Next, we visualized the strong enrichment of statistical association with cranial vault shape amongst SNPs associated with brain and facial shape using conditional Q-Q plots^54^ (Fig. 1e, f). Under the global null hypothesis, the nominal distribution of *P* values from any GWAS is expected to follow a uniform distribution, represented by the diagonal line. An increase in tail probabilities due to true genetic associations with cranial vault shape presents itself as a deflection from the expected diagonal line in the Q-Q plots. Here, we observed that the deflection was exacerbated (i.e. shifted leftward) when conditioning on the *P* values from the brain or facial shape GWAS, i.e., using the *P* values from those GWASs to define subsets of SNPs from the cranial vault GWAS, indicating an increase in the number of SNPs across the genome showing evidence of association with cranial vault shape. Notably, the magnitude of this leftward shift correlated with the strength of association with brain or facial shape. Previous work by Andreassen et al.^54^ demonstrated that such a leftward shift serves as a conservative measure for the FDR of genotype-phenotype associations, with a more pronounced shift indicating a lower FDR.

Building on this, we applied the conditional FDR method^54^, leveraging the strength of a SNP’s association with auxiliary phenotypes as prior information, or evidence to reduce the prior, or unconditioned FDR of its association with cranial vault shape (Fig. 1g). The heatmaps demonstrate how a SNP’s *P* value (denoted as the minus logarithm) from the cranial vault shape GWAS can be combined with its *P* value for an auxiliary phenotype to obtain a posterior, or conditional FDR (cFDR). The 1% cFDR boundary in each heatmap, exhibits a saturating behavior, i.e., it approaches a vertical asymptote located at a non-zero minus-log value of the cranial vault *P* value. This demonstrates that irrespective of the conditional trait, a minimal association in the cranial vault GWAS is necessary for a SNP to attain 1% cFDR. Comparing the 1% cFDR boundaries across auxiliary traits revealed that facial shape and brain shape most effectively reduced this minimal requirement, better than BMD and height (Fig. 1g). Altogether, the results presented in this section clearly demonstrate that the genetic architectures of facial shape, brain shape, and BMD are enriched for associations with cranial vault shape and consequently, that GWAS data on those traits can provide additional evidence for SNPs associated with cranial vault shape.

### Pleiotropy-informed GWAS of cranial vault shape boosts genetic discovery

We conducted a pleiotropy-informed GWAS analysis of cranial vault shape using the conditional FDR method^54^, hereafter referred to as cFDR-GWAS, by conditioning a recent cranial vault shape GWAS^6^ on one of three auxiliary GWAS datasets: brain shape (“Vault | Brain”), facial shape (“Vault | Face”), or BMD (“Vault | BMD”). Analogous to the previous section, conditioning in this context refers to leveraging auxiliary GWAS data as evidence in an empirical Bayesian framework where the conditional test statistic can be formally defined as the conditional probability (i.e., the Bayesian posterior probability) that a SNP is null for cranial vault shape given that its *P* values for cranial vault shape and the auxiliary phenotype are equal to or smaller than the observed *P* values (“Methods”). This probability can be interpreted as the empirical Bayesian generalization of the FDR^74^.

**Fig. 2:**
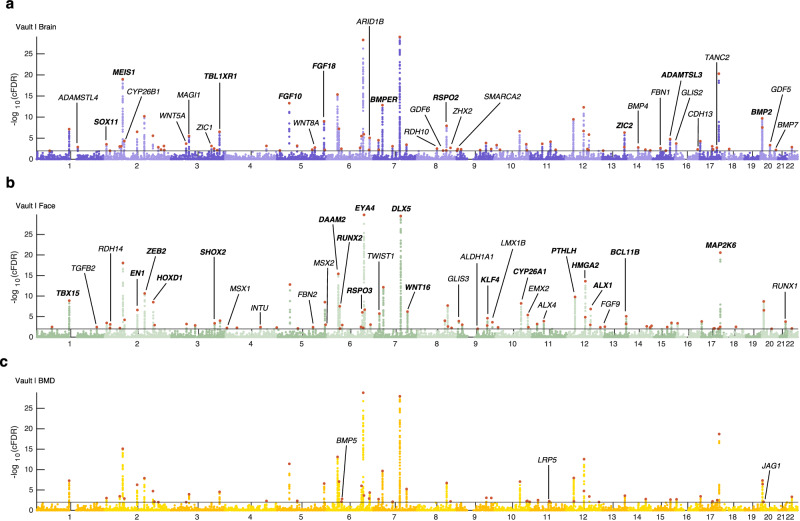
Pleiotropy-informed GWAS of cranial vault shape improves genetic discovery. Manhattan plots for cFDR-GWAS of cranial vault shape, conditioned on GWAS data on **a** brain shape (purple, “Vault | Brain”), **b** facial shape (green, “Vault | Face”), and **c** heel bone mineral density (yellow, “Vault | BMD”). Horizontal lines indicate the 1% cFDR threshold. Lead SNPs are indicated in red. Candidate genes at a selection of loci are indicated on the Manhattan plot where the lowest cFDR was obtained for that locus. Loci that were previously mentioned in GWAS on cranial vault dimensions are indicated in bold.

**Fig. 3:**
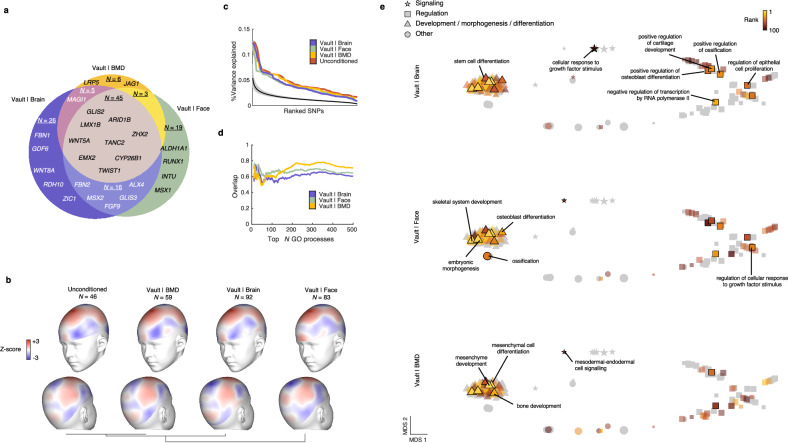
Pleiotropy-informed GWAS of cranial vault shape yields consistent results across auxiliary traits. **a** Venn diagram showing locus overlap between the cFDR-GWAS analyses in Fig. 2. Venn diagram dimensions are proportional to the number of loci. A selection of genes not previously mentioned in cranial vault GWAS literature is shown. **b** Patterns of variance explained by the set of lead SNPs from each cFDR-GWAS. The vertex-wise variance explained was transformed into Z-scores for each cFDR-GWAS independently. Towards red indicates high explained variation by the lead SNPs of each respective GWAS. Vertex-wise patterns of Z-scores were clustered based on pairwise correlations. **c** Distributions of the variance explained by individual lead SNPs of each GWAS. Solid black line and gray band give the mean and 95% confidence interval for the null estimate through 1000 random genotype permutations. **d** Proportional overlap between the sets of the top N most enriched GO biological processes obtained for the cFDR-GWASs and that obtained for the unconditioned GWAS. **e** Semantic space of the joint set of GO biological processes that were significantly enriched (at 5% FDR) amongst the loci from each individual cFDR-GWAS in Fig. 2. The space was obtained using REVIGO based on multidimensional scaling (MDS) of the pairwise similarities between GO biological processes in the set. Different symbols indicate different broad categories of processes, and symbol size corresponds to the total number of genes for each process. Corresponding to each cFDR-GWAS, the top 100 most enriched processes are indicated and colored by their rank. A selection of processes is annotated in the plot where the process attained the lowest rank. Lead SNPs for the “unconditioned” GWAS in (**b–d**) were called at 1% FDR to provide a fair comparison.

As demonstrated above, conditioning on these three traits resulted in the greatest enrichment of statistical association with cranial vault shape. However, there are notable distinctions among these traits. Firstly, while brain shape is not traditionally considered a skeletal trait, it is structurally integrated with the cranial vault and explains a significant portion of its overall shape variance. Secondly, facial shape primarily reflects skeletal features and is predictive for certain aspects of cranial vault morphology (e.g. the forehead region). On the other hand, BMD serves as a systemic skeletal trait which contains limited information about the shape of the cranial vault. Nonetheless, our analysis revealed remarkably similar genome-wide association profiles across all three cFDR-GWAS datasets (Fig. 2), underscoring the robustness of the findings. In total, we identified 120 genomic loci associated with cranial vault shape at a 1% cFDR in at least one of the cFDR-GWAS analyses (Fig. 2a, Supplementary Data 1), a marked increase compared to the 46 loci identified at 1% FDR in the unconditioned GWAS. The expected number of false positive loci at the 1% cFDR cutoff was estimated conservatively as 0.54, ensuring reliable results at this threshold. In total, 90 of the 120 identified loci were not previously mentioned in GWAS of cranial vault morphology. Of these, 75 (83.3%) discoveries can be attributed to the conditional approach, while 15 (16.7%) were simply due to setting the threshold at a 1% cFDR instead of *P* < 5e–8. Notably, the cFDR-GWAS analyses that leveraged brain and facial shape data yielded a higher number of genomic loci (*n* = 92 and *n* = 83 respectively) compared to the one using BMD data (*n* = 57) in line with the above results (Fig. 1c). These loci strongly overlapped across all three cFDR-GWAS analyses (Fig. 3a). Furthermore, among the 51 loci identified exclusively in a single cFDR-GWAS at a 1% cFDR threshold, 28 (54.9%) also achieved a cFDR <5% in at least one of the other analyses (Supplementary Data 1).

We investigated the patterns of cranial vault shape variation explained by the lead SNPs identified in both the unconditioned and the cFDR-GWASs of cranial vault shape. Remarkably, these patterns exhibited high concordance across all GWAS analyses, with the greatest variance explained in regions near the Bregma point and the parietal eminence (Fig. 3b). Unsurprisingly, lead SNPs from the cFDR-GWAS based on facial shape excelled in explaining variation across the forehead, while lateral forehead regions remained among the least explained by lead SNPs from other GWASs (Fig. 3b). As a result, the pattern of cranial vault shape variation explained by the cFDR-GWAS based on facial shape was the most dissimilar to that of the unconditioned GWAS, whilst that of the cFDR-GWAS based on BMD was the most similar. In terms of overall shape variation explained by individual lead SNPs of the unconditioned and cFDR-GWAS analyses, similar distributions were observed (Fig. 3c). When combining all the lead SNPs, the unconditioned GWAS explained 2.02% of the overall cranial vault shape variation. This percentage increased to 3.38%, 2.98%, and 2.32% for the lead SNPs from the cFDR-GWASs based on brain shape, facial shape, and BMD respectively, with a cumulative explanation of 4.11% by the combined set of 120 lead SNPs. Despite this substantial overall increase, the patterns of explained shape variation by the cFDR-GWAS lead SNPs remained remarkably similar to the unconditioned GWAS, suggesting that the additional loci exert similar effects on the cranial vault as those identified previously.

We then explored whether the loci identified in each GWAS (at a 1% cFDR) consistently implicated the same biological processes. To accomplish this, we utilized GREAT^75^ to annotate Gene Ontology (GO) biological processes to both the unconditioned GWAS and each cFDR-GWAS, tracking the number of overlapping terms (Fig. 3d). Overall, each cFDR-GWAS revealed GO biological processes that strongly overlapped with those identified in the unconditioned GWAS. To further investigate the consistency of the terms identified across each cFDR-GWAS, we aggregated the significant terms (at 5% FDR) into a union set and constructed a single joint semantic space using REVIGO^76^. In this semantic space, Multidimensional Scaling (MDS) clustered closely related terms, revealing clusters broadly associated with development, signaling, and regulation (Fig. 3e). In this space, we then indicated the top 100 terms from each cFDR-GWAS separately. While it should be expected that each cFDR-GWAS yields a slightly different set of processes resulting from the identification of unique loci, Fig. 3e demonstrates that these unique processes remain closely related to those identified by the other cFDR-GWAS. In essence, while conditioning on different auxiliary phenotypes led to partially overlapping sets of loci, the choice of secondary phenotype did not result in divergent biological findings.

### Pleiotropy-informed GWAS implicates key signaling pathways in craniofacial development

**Fig. 4:**
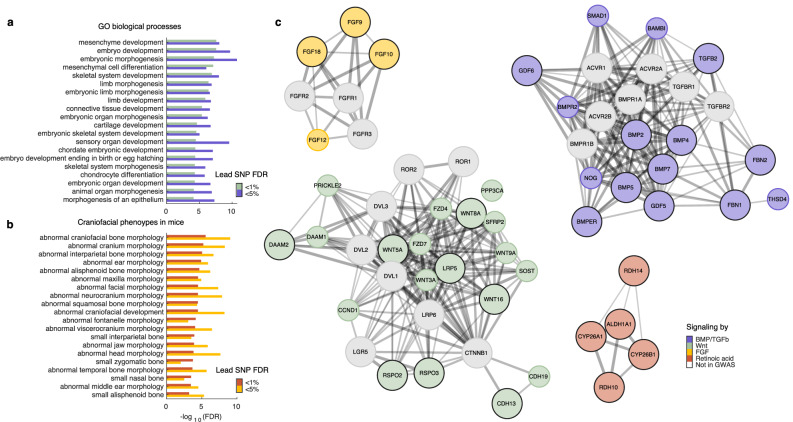
Pleiotropy-informed GWAS implicates major signaling pathways involved in craniofacial development. **a** Enrichment of GO biological processes amongst cFDR-GWAS loci identified at 1% and 5% FDR. Only the top 20 processes are shown. **b** Enrichment of mouse phenotypes amongst cFDR-GWAS loci identified at 1% and 5% FDR. Only the top 20 craniofacial phenotypes, selected from the full list of phenotypes, are shown. **c** STRING networks representing members of the BMP/TGF-β, Wnt, Retinoic acid, and FGF signaling pathways identified in the cFDR-GWAS. Genes in large, colored nodes with a black border were identified at 1% cFDR; those in smaller, colored nodes without a black border were identified at 5% cFDR; and genes in white nodes were not identified in the GWAS but are added to provide context.

The use of FDR-based statistics in GWAS allows for straightforward control of the number of falsely identified loci. While it is common practice in GWAS to use a very strict significance threshold to declare positive associations, we reasoned that relaxing the threshold to 5% cFDR (instead of 1% cFDR) would yield a larger set of genomic loci, still enriched for biologically meaningful information^77^, that could help to more robustly implicate biological processes and pathways. Therefore, to further explore the SNPs and genes underlying cranial vault shape, we lowered the significance threshold to 5% cFDR in each cFDR-GWAS and subsequently merged their genomic loci into 328 independent loci (Supplementary Data 1), explaining 8.53% of overall cranial vault shape. Following an enrichment analysis using GREAT^75^, we observed, indeed, that most of the top 20 GO biological processes and the top 20 craniofacial mouse phenotypes identified for the initial set of 120 loci were consistently and more strongly supported among the broader set of 328 loci (Fig. 4a, b; Supplementary Data 2–5). Furthermore, among the loci identified at a 5% cFDR, we identified additional genes linked to craniosynostosis in humans, including *PPP3CA*^78^, *CDKN1*C^79^, *FOXP1*^80^*, PRRX1*^80^, and *ZBTB20*^80^. Taken together, these results show that relaxing the significance threshold to 5% cFDR helped strengthen the support for key biological processes and pathways through the identification of additional, biologically meaningful candidate genes.

Among the genes near the identified loci were many members of the Wnt, BMP/TGF-β, and FGF signaling pathways as well as several genes related to metabolism of retinoic acid (Fig. 4c), which itself is a key signaling molecule during craniofacial development. We found a wide variety of genes involved in both beta-catenin dependent and independent Wnt signaling including ligands (*WNT3A*, *WNT5A*, *WNT8A*, *WNT9A*, *WNT16*, *RSPO2*, and *RSPO3*), antagonists (*SOST* and *SFRP2*), (co-)receptors (*FZD4*, *FZD7*, and *LRP5*), and other genes related to signal transduction (*PRICKLE2*, *DAAM1*, *DAAM2*, *CCND1*, and *PPP3CA*). Furthermore, we identified several *fibroblast growth factors* (FGFs) with known roles during craniofacial development, including *FGF9*, *FGF10*, *FGF12*, and *FGF18*. Notably, none of their receptors were identified despite their prominent roles in cranial vault development as evidenced by their causative roles in craniosynostosis^81^. From the BMP/TGF-β signaling pathway, we identified several extracellular signaling ligands, including *growth differentiation factors* (GDFs; *GDF5* and *GDF6*), *bone morphogenic proteins* (BMPs; *BMP2*, *BMP4*, *BMP5*, and *BMP7*), and *TGFB2*, as well as one BMP-receptor (*BMPR2*), signaling inhibitors (*NOG* and *BAMBI*), and modulators of BMP/TGF-β signaling (*BMPER*, *FBN1*, *FBN2*, and *THSD4*). Lastly, we identified several genes related to the biosynthesis (*ALDH1A1*, *RDH10*, and *RDH14*) and degradation (*CYP26A1*, and *CYP26B1*) of retinoic acid.

### Cross-trait associations are specifically enriched at mesenchymal TF targets

**Fig. 5:**
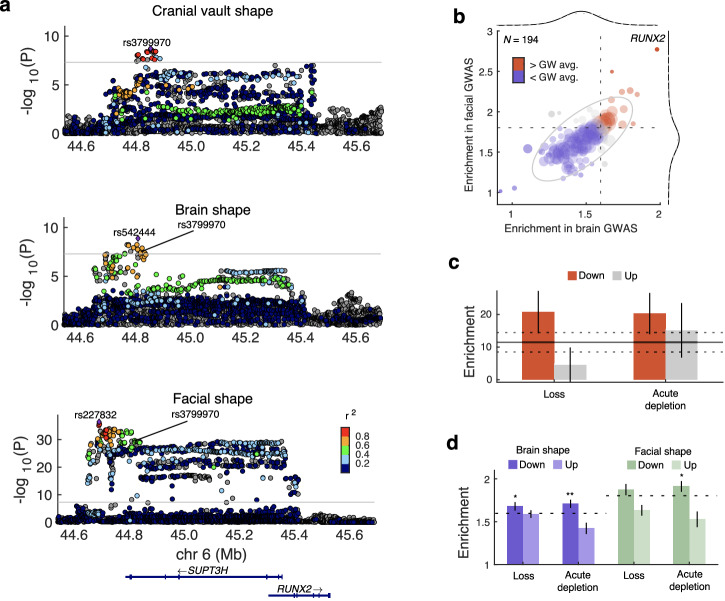
Cross-trait associations with cranial vault shape are specifically enriched at binding sites of mesenchymal transcription factors. **a** LocusZoom plots show locus around rs3799970, near RUNX2 in the cranial vault, brain, and facial shape GWAS. Colors represent the LD (r^2^; 1000G EUR populations) with the lead SNP from each GWAS. SNPs in gray did not overlap with 1000G reference set. The horizontal line indicates the *P* < 5e–8 threshold. **b** Biplot showing fold-enrichment of statistical associations between cranial vault shape and brain or facial shape, but specifically for the SNPs located in the binding domain of various TFs (*N* = 194 + RUNX2). Dashed lines indicate the genome-wide estimate which is the same as in Fig. 1c. Empirical distributions (gray dashed lines) on top of fitted t-distributions (black full lines) are show for both axes. The ellipse indicates the empirical 95% confidence boundary. **c** Fold enrichment of cranial vault shape heritability (from Goovaerts et al.^6^) in distal ATAC peaks differentially accessible upon TWIST1 depletion (*N*_up_ = 15,727; *N*_down_ = 35,553) or loss (*N*_up_ = 38,236; *N*_down_ = 31,689). Error bars indicate the standard error. The horizontal line indicates the enrichment in all human CNCC distal ATAC peaks (*N* = 189,601), with flanking dashed lines the standard error. **d** Fold-enrichment of statistical associations between cranial vault shape and brain (purple) or facial shape (green), but specifically for the SNPs located at distal ATAC peaks differentially accessible upon TWIST1 depletion or loss. Error bars indicate standard errors estimated over 1000 bootstrapped sets of peaks. Dashed horizontal lines indicate the genome-wide estimate which is the same as in Fig. 1c. (*P < 0.05, **P < 0.01, one-tailed *t* test).

Our previous GWAS on cranial vault shape identified a locus near *RUNX2*, encoding an osteogenic transcription factor (TF) which is expressed in the craniofacial mesenchyme, but not in the brain. Nonetheless, we found that this locus overlaps with brain shape^33^ and facial shape (most strongly with the nose)^7^ (Fig. 5a). The lead SNPs from the cranial vault shape (rs3799970) and brain shape (rs542444) GWASs are in near perfect linkage (D’ = 0.994; r^2^ = 0.792; 1000 G EUR populations), affirming that this is the same locus. The facial shape lead SNP, rs227832, was located ~130 kb away from rs3799970, but still in strong linkage (D’ = 0.889; r^2^ = 0.500; 1000 G EUR populations). Next, we examined if the binding sites of RUNX2 were specifically enriched for cross-trait associations between the cranial vault and either the brain or face, similarly to Fig. 1c. To this end, we obtained binding site coordinates for RUNX2 and 194 other TFs from TFLink^82^ and calculated the cross-phenotype enrichments across the binding sites of each TF. Brain-associated and face-associated SNPs at RUNX2 binding sites were significantly more enriched for vault-associated SNPs (Fig. 5b and Supplementary Data 6;1.98-fold and 2.77-fold respectively) compared to those at other TF’s binding sites (*P*_brain_ = 1.75e–4; *P*_face_ = 2.15e–7; one-tailed *t* test). This suggests that activity of the osteogenic TF, RUNX2, underlies in part the cross-trait enrichment of associations.

Despite its well-known role in craniofacial skeletal development and its causative role in syndromic craniosynostosis, TWIST1 was not implicated by any GWAS of cranial vault dimensions previously. However, by following a pleiotropy-informed GWAS approach, we did identify a locus near *TWIST1*. Using stratified linkage disequilibrium score regression (S-LDSC) on the summary statistics from our previous cranial vault shape GWAS^6^, we examined if genomic targets regulated by TWIST1 were disproportionately enriched for heritability. From a previous publication^83^, we obtained the coordinates of distal assay for transposase accessible chromatin (ATAC) peaks that were differentially accessible upon loss or acute depletion of TWIST1 in human CNCCs. Figure 5c shows how the down-regulated TWIST1-dependent peaks were specifically enriched for cranial vault shape heritability (TWIST1 loss: 20.7-fold, *P* = 6.87e–4; and TWIST1 depletion: 20.3-fold; *P* = 1.25e–3, one-tailed *t* test). It was previously shown^83^ that the down-regulated, but not the upregulated peaks were highly enriched for TWIST1 binding motifs. Therefore, these findings provide orthogonal validation that regulation by TWIST1 plays a role in typical-range cranial vault shape variation.

Furthermore, SNPs at the downregulated TWIST1 peaks were also more enriched for associations with cranial vault shape compared to the genome-wide estimate (Fig. 5d), both in the brain shape GWAS (TWIST1 loss: 1.68-fold, *P* = 0.0495; TWIST1 depletion: 1.71-fold, *P* = 8.75e–3; one-tailed *t* test) and facial shape GWAS (TWIST1 depletion: 1.91-fold, *P* = 0.0387; one-tailed *t* test). Even after Bonferroni-correction, the 1.71-fold enrichment of vault-associated SNPs among the brain-associated SNPs at down-regulated peaks upon acute TWIST1 depletion was still significantly higher than the genome-wide estimate of 1.59 (*P*_adj_ = 0.0350; one-tailed *t* test). Together, these analyses demonstrate that SNPs located at the binding sites of mesenchyme-specific TFs contribute strongly to the cross-trait genetic enrichment between the cranial vault and both the face and brain. These findings align with the strong enrichments of mesenchyme-related processes among the genes located near the cFDR-GWAS loci.

## Discussion

In this work, we utilized a pleiotropy-informed approach to enhance the detection of SNPs associated with cranial vault shape and to deepen our genetic understanding of this understudied component of the craniofacial system. The conditional FDR method, inspired by the empirical Bayes framework, states that the FDR of genotype-phenotype associations may be reduced by incorporating prior information on the SNPs as evidence, specifically regarding whether they have prior associations with closely related traits. Here, we first demonstrated that SNPs associated with traits closely related to cranial vault shape are indeed more likely to be associated with it. We then conditioned a recent cranial vault shape GWAS^6^ on the *P* values from a facial shape^7^, brain shape^33^, and bone mineral density^65^ GWAS, yielding 120 independent loci at a 1% cFDR, and 328 at a 5% cFDR, substantially more than previously identified^6,22–27^.

Conditioning on GWAS data from three distinct phenotypes was proven valuable not only to improve genomic discovery, but also in demonstrating the robustness of our findings. Specifically, our analyses revealed remarkably consistent genome-wide association profiles, biological processes, and phenotypic effects across all cFDR-GWASs. This consistency underscores the robustness of the genetic associations identified and suggests that the observed associations between genetic variants and cranial vault shape are not solely influenced by the specific traits used for conditioning. In fact, these findings illustrate that cross-phenotype genetic associations are abundant among different constituents of the craniofacial system and bone-related measurements more broadly and that they could effectively be leveraged to boost GWAS discovery. This aligns with the widespread pleiotropy of craniofacial transcription factors, genes involved in system-wide skeletal development, and the mutual influences between the face, brain, and cranial vault that occur on structural and physiological levels during head development^1,11,33^. Still, we note a few examples where the choice of auxiliary trait was directly related to the findings. Only when leveraging BMD, we identified a locus near *LRP5* at a 1% cFDR, for which loss-of-function mutations are associated with osteoporosis and gain-of-function mutations with higher bone mass and craniosynostosis^84,85^. Additionally, a locus near *MSX1* was only identified at a 1% cFDR by leveraging facial shape. This gene plays a key role in facial development, including the frontal bone^86^, with mutations linked to multiple craniofacial anomalies such as cleft lip and palate^87^.

Only after the forebrain initially forms from the rostral end of the neural tube do cranial neural crest cells and mesodermal progenitor cells give rise to much of the craniofacial skeleton^8,10,88^. From that point onward, neurological and mesenchymal tissues develop with tight coordination^1,11^. Here, we have demonstrated how a substantial proportion of cranial vault shape information is embedded in the brain’s shape as a product of this co-development and how the regulatory landscapes of mesenchymal TFs are enriched for their cross-trait genetic associations. Together, these findings suggest that brain shape, in part, reflects the development of the craniofacial skeleton and consequently, that brain-derived traits subjected to GWAS may implicate cranial vault-associated loci. In fact, examples of such loci have been reported previously^6,33^, including *RUNX2*, *TWIST1*, and *ALX1*, which are not expressed in the brain but play prominent roles in craniofacial skeletal development^28,83^. More generally, several brain shape GWAS studies have found skeletal processes to be overrepresented among the identified genes^33,44^. These findings could arise from mediated pleiotropy^89^, where a typically well-powered brain GWAS picks up on the skeletogenic genes that affect brain shape through their effects on the cranium. Here, it is likely that mediated pleiotropy underlies, at least partially, the many cross-phenotype genetic associations between the brain and cranial vault and therefore the success of the conditional FDR method.

Both the vicerocranium and the vault portion of the neurocranium form through intramembranous ossification, i.e., the direct ossification of mesenchymal cells^88^. This process is regulated by osteogenic transcription factors, such as RUNX2 and SP7, as well as extracellular signaling by FGF, BMP, Wnt, and IHH^29,90^. Additionally, many of these genes affect bone mineral density across the body through processes including bone formation, homeostasis, and remodeling^91,92^. Moreover, craniofacial transcription factors from the *basic helix-loop-helix* and *homeodomain* families cooperatively control the development of neural crest-derived mesenchyme in the face and cranial vault^83,93^. This high degree of biological pleiotropy underlies the genomic overlap between these skeletal phenotypes and therefore contributes to the effectiveness of the conditional FDR method. Additionally, since the definitions of the cranial vault and face in the original GWASs partially overlap at the forehead, any locus affecting that area would automatically be associated with both phenotypes but would not always be detected if statistical power is low. This likely constitutes a secondary reason why leveraging facial shape was effective in a cFDR-GWAS.

Our pleiotropy-informed GWAS identified a plethora of loci near genes with well-known roles in cranial vault development. Most notably, we identified a locus near *SP7* and two loci near *RUNX2*, which encode transcription factors essential for osteoblast differentiation and bone formation^94^. In addition, many of the other genes identified have direct or indirect effects on the activity of *SP7* and *RUNX2*. For example, signaling by BMPs and IHH are known to induce expression of *SP7* in a manner dependent on RUNX2, or independent of RUNX2 through MSX2^95–99^. Meanwhile, reciprocal induction of *RUNX2* expression and signaling by Wnt, FGF, and PTHLH allow for precise control of osteogenesis^30,31,90^. Mutations in several of these genes have been linked to craniosynostosis, i.e., the premature fusion of one or more cranial sutures, a condition that affects approximately 1/2500 births^100^. Interestingly, genes that likely induce *RUNX2* or *SP7* expression tend to cause craniosynostosis through gain-of-function mutations or whole gene duplications, such as *MSX2*^101,102^, *LRP5*^85^, *SOX11*^103^, *IHH*^104^, and *RUNX2* itself^105,106^. The opposite phenotype is observed for deletions in *MSX2*^107^ or *RUNX2*^108^, suggesting a dosage-sensitive effect of both genes on cranial suture fusion^81^. Along the same lines, loss-of-function mutations in *TWIST1*, which directly antagonizes RUNX2 in the developing coronal suture, cause Saethre-Chotzen syndrome, characterized by coronal craniosynostosis^109,110^. Given that the genes near many of the loci identified in our pleiotropy-informed GWAS are directly or indirectly related to *RUNX2* or *SP7*, it is possible that together they can predict a considerable proportion of craniosynostosis risk. In fact, GWASs on non-syndromic craniosynostosis have identified risk SNPs near *BMP2*, *BMP7*, *BMPER*, and *DLX5*, which overlap with loci identified here, and are directly linked to *RUNX2*^6,111–115^. Due to their large effect sizes, these loci could be identified in relatively small patient cohorts, however, it is likely that many more loci are involved in non-syndromic craniosynostosis risk, such as other loci identified here. Furthermore, recent reports have noted that normocephalic sagittal craniosynostosis is underdiagnosed and therefore more prevalent than previously thought, with estimates as high as 4.7% in the overall population^116,117^. Consequently, our pleiotropy-informed GWAS may pick up on craniosynostosis-related signals directly. In addition to contributing risk, the polygenic background comprised of the identified loci may be predictive for phenotype severity, an idea already demonstrated in mice^118^.

In addition, we identified genes involved in the two-step oxidation of retinol (vitamin A) into the bioactive retinoic acid. Specifically, *RDH10* and *RDH14* encode enzymes that oxidize retinol into retinaldehyde^119,120^, which is further oxidized into retinoic acid by ALDH1A1^119^. Previous studies have found that retinoic acid interacts with the Wnt, Hedgehog, and BMP pathway during osteogenesis^121,122^, whereby excess retinoic acid causes skeletal anomalies that are phenocopied by loss-of-function mutations in *CYP26A1* and *CYP26B1*, which encode enzymes involved in retinoic acid degradation^119,123,124^. Notably, retinoid-induced craniosynostosis has been observed in the case of homozygous mutations in *CYP26B1*^124–126^. These findings suggest that genes involved in regulating retinoic acid levels during embryonic development contribute not only to craniofacial anomalies, but also to the variation in cranial vault shape seen in the general population. Since maternal diet affects embryonic retinoid availability^127^, retinoid-related effects on cranial vault shape are likely, to some extent, mediated by gene-environment interactions.

As with other GWAS studies, replication of the genetic findings is key to confirm their validity. Unfortunately, the limited availability of linked craniofacial and genetic data poses significant challenges for both genetic discovery and replication efforts, especially for the cranial vault. While 3D facial data can be collected through 3D surface scanning, this is much more difficult to achieve for the cranial vault due to the presence of hair. Alternatively, medical imaging provides a 3D view of the vault, but has other limitations, e.g., computed tomography (CT) imaging exposes participants to radiation and MR-imaging is expensive. A workaround is to use existing brain MR-image collections to extract the cranial vault^6^, a task that poses technical challenges since imaging protocols are optimized regarding the brain. Moreover, due to privacy concerns, these images are often anonymized by removing parts of the face and ears, regions that overlap with the cranial vault^128^. While additional data sources and larger sample sizes are necessary to further elucidate the genetic architecture of complex traits in general, multi-trait GWAS strategies already enhance genetic insights in the meantime^54,129–131^. In this work, we specifically opted for the conditional FDR method as it could seamlessly deal with the multivariate GWAS statistics of cranial vault shape and the other morphological phenotypes. We opted for a threshold of 1% cFDR to call statistical significance of individual loci, whereby fewer than one locus among the 120 independent loci is expected to be a false positive. At 5% cFDR, the expected number of false discoveries was still low, hence, we used these loci to implicate additional candidate genes related to the already identified pathways. This level of confidence may be too low to warrant the time and cost-intensive functional follow-up of individual loci. However, collectively, our loci may form the basis for candidate gene studies or polygenic risk scores (PRS) in small cohorts of patients with craniofacial conditions, e.g., craniosynostosis, where they could directly lead to enhanced etiological understanding and risk prediction.

In summary, the conditional FDR method allowed the use of prior knowledge to inform on genetic associations with human cranial vault shape, resulting in an enhanced discovery of SNPs. The identified genes robustly implicate the Wnt, BMP/TGF-β, FGF, and retinoic acid pathways to play key roles in shaping the vault. Given the close link between many of the identified loci and the master regulators of suture ossification, *RUNX2* and *SP7*, it is plausible that collectively our loci comprise a polygenic background predictive of craniosynostosis risk and severity. In sum, our improved GWAS discovery substantially enhances the genetic understanding of typical-range variation in cranial vault shape and craniofacial development more broadly.

## Methods

### Ethics statement

Data available through the controlled access NIMH data archive has been approved for broad sharing. Local institutional approval (S60568) was granted for access to this data. Legal guardians of ABCD study (https://abcdstudy.org/about/) participants provided written informed consent to participate and have their data shared.

### Cohort data

For investigations into the proportion of explainable vault shape variation by genetic and phenotypic variables, we used participant data from the ABCD Study, a 10-year longitudinal study following brain health and development through adolescence^63^. A variety of data from 11,880 nine- and ten-year-old boys and girls has been collected across 21 sites in the US and have been made available through the controlled-access NIMH data archive (https://nda.nih.gov/). The data used in this work, including T1-weighted MRIs, genotypes, and additional information on sex at birth, age, weight, height, etc. were obtained from data release 3.0 (February 2020).

### GWAS summary data

GWAS summary statistics were obtained from our recent cranial vault shape GWAS^6^ and from recent, large-scale GWAS studies^7,33,65–73^ on other complex phenotypes conducted in independent cohorts of predominantly recent European ancestry. This includes the cranial vault GWAS in the ABCD cohort, for which ~80% of the alleles were derived from recent European ancestors^6^, which forms the basis for the main analyses performed in this work. Additionally, a version of this GWAS, conducted in a sub-sample with inferred European ancestry, was used only for S-LDSC. All GWAS data used in this work are freely available online. An overview of studies and sample sizes is given in Supplementary Table 1.

### Image processing and phenotyping

From the ABCD study’s T1-weighted MR images, we extracted the mid-cortical surface using FreeSurfer v6.0.0^132^, as well as the facial and cranial vault surface using our previously described pipeline^6,62^ and Meshmonk^133^. Only participants with inferred recent European ancestry were retained as abundant imaging artifacts can disproportionately affect individuals of different ancestries due to differences in hair types and facial structures, increasing the risk of spurious results. A detailed description of image processing and sample selection can be found in the Supplementary Note and Supplementary Fig. 2.

Quality controlled 3D surface meshes of the brain (*n* = 29,759 vertices), face (*n* = 7160 vertices), and cranial vault (*n* = 11,410 vertices) were Procrustes superimposed separately and then bilaterally symmetrized. After removing a set of related individuals up to the third degree using KING^134^ (cutoff = 0.0442), the resulting surfaces were adjusted for covariates, including sex at birth, age, weight, height, site/MRI machine, brain/vault/facial size, and genomic ancestry using partial least squares regression (Matlab 2023a, *plsregress*). Due to the abundance of facial artifacts, facial surfaces were additionally adjusted for artifact-related variables (Supplementary Note and Supplementary Fig. 2) and their interaction terms with BMI, head size and facial size. Finally, we extracted the optimal number of variables to describe the phenotypes using PCA and parallel analysis on all three phenotypes separately.

### Cross-phenotype explained variance

Using partial least squares regression (Matlab 2023a, *plsregress*) and data from 1969 individuals with high quality brain, facial, and cranial vault data available, we estimated the percentage of cranial vault shape variation explained in each principal component by the full set of principal components from either the brain or face. The final PCA models contained 37, 46, and 286 principal components for the vault, face, and brain, explaining 95.7%, 96.7%, and 77.1% of their morphological variation respectively.

### Genomic Spearman correlations

Unlike most GWASs on univariate traits, the multivariate GWAS on cranial vault shape did not yield signed effect sizes. Therefore, the commonly used approach of calculating genomic correlation using linkage disequilibrium (LD) score regression^135^ was not applicable here and instead we opted to calculate genomic Spearman correlations as described by Naqvi et al.^33^. Briefly, SNPs were intersected with the HapMap3^136^ set of SNPs excluding any SNP within the major histocompatibility region (GRCh37 positions of chr6:25,119,106–33,854,733). The remaining SNPs were then organized into 1725 approximately independent LD blocks^137^ estimated in European populations^138^. We used European-derived LD blocks since all GWAS data was generated in samples of predominant recent European ancestry.

The genomic Spearman correlation (Matlab 2023a, *corr* with parameter ‘*Spearman*’) between both traits and using $n$ LD blocks was calculated based on Eq. 1, where ${d}_{i}\equiv R[\bar{-{\log }_{10}({P}_{i,x})}]-R[\bar{-{\log }_{10}({P}_{i,y})}]$, the difference in rank between the average $-{\log }_{10}\left(P\right)$-value of traits $x$ and $y$ in LD block $i$.

1 $${r}_{g}(x,y)=1-\frac{6\mathop{\sum }_{i=1}^{n}{d}_{i}}{n\left({n}^{2}-1\right)}$$

### Cross-trait enrichment of statistical association

The enrichment of statistical association with cranial vault shape among the SNPs associated with another trait was defined as the fold increase in the proportion of SNPs associated with cranial vault shape (at *P*_*vault*_ < 0.05) among the SNPs associated with an auxiliary trait (at *P*_*other*_ < 0.05) relative to the set of all SNPs (Eq. (2)). The resulting fold-enrichment essentially measures the increase in tail-probabilities in the *P* value distributions of the subset of SNPs relative to the full set. By Bayes’ theorem, it follows that this definition of the cross-trait fold-enrichment is reciprocal (Eq. (2)), however, this property is not further utilized in this work.

2 $${fold}\; {enrichment} = \, \frac{{Prob}({P}_{{vault}} < {0.05} \, | {P}_{{other}} < {0.05})}{{Prob}({P}_{{vault}} < {0.05})}= \,\frac{{Prob}({P}_{{other}} < {0.05}\, |{P}_{{vault}} < {0.05})}{{Prob}({P}_{{other}} < {0.05})}$$

Similarly, the cross-trait enrichment specific to a TF, *t*’s binding sites, *B*_*t*_, was obtained by conditioning the probabilities in Eq. (2) on the prerequisite that a SNP, *s*, is in *B*_*t*_ (Eq. (3)).

3 $${fold}\; {enrichment} = \,\frac{{Prob}\left({P}_{{vault}} < {0.05}|{P}_{{other}} < {0.05},\,s\in {B}_{t}\right)}{{Prob}\left({P}_{{vault}} < {0.05}\,|\,s \in {B}_{t}\right)}$$

Binding sites for various TFs (*N* = 194 + RUNX2, after excluding TFs with <500 binding sets across the autosome) were obtained from TFlink^82^ v1.0 (https://cdn.netbiol.org/tflink/download_files/TFLink_Homo_sapiens_bindingSites_All_annotation_v1.0.tsv.gz). The binding sites of RUNX2 were obtained through a chromatin immunoprecipitation (ChIP) assay in osteosarcoma cells^139^. Distal hCNCC ATAC peaks differentially accessible upon TWIST1 loss or acute depletion were obtained from Kim et al.^83^. In both cases, we selected all SNPs within a margin of 10 kb around each binding site or peak.

### Conditional FDR analysis

As originally introduced by Andreassen^54^, the conditional FDR of a genotype-phenotype association is defined as the conditional probability (i.e., the Bayesian posterior probability) that a SNP is null for the target phenotype given that the *P* values for the target and auxiliary phenotype are equal to or smaller than the observed *P* values. Formally, using the cranial vault as the target phenotype, this can be written as

4 $${FDR}\left({P}_{{vault}}|{P}_{{other}}\right)=\frac{{\pi }_{0}\left({P}_{{other}}\right){F}_{0}\left({P}_{{vault}}|{P}_{{other}}\right)}{F\left({P}_{{vault}}|{P}_{{other}}\right)}$$

where ${F}_{0}$ and $F$ are the conditional cumulative density functions (CDF) for null and all SNPs respectively, and ${\pi }_{0}\left({P}_{{other}}\right)$ is the proportion of SNPs that are null for the cranial vault given that the *P* value for the auxiliary phenotype is equal to or smaller than ${P}_{{other}}$. Assuming independence, ${F}_{0}\left({P}_{{vault}},|,{P}_{{other}}\right)$ simplifies to ${P}_{{vault}}$. Moreover, to estimate ${FDR}\left({P}_{{vault}}|{P}_{{other}}\right)$ conservatively, ${\pi }_{0}\left({P}_{{other}}\right)$ is set to 1 and $F\left({P}_{{vault}}|{P}_{{other}}\right)$ is replaced with the empirical conditional CDF. This formulation is a generalization of the empirical Bayesian interpretation of the FDR by Efron^74^.

The conditional FDR analysis was conducted based on the *pleioFDR*^54^ software (https://github.com/precimed/pleiofdr). Due to complex Linkage Disequilibrium (LD) that can potentially bias FDR estimates, SNPs within the major histocompatibility region on chromosome 6 and chromosome 8p23.1 (GRCh37 positions of chr6:25,119,106–33,854,733 and chr8:7,200,000–12,500,000, respectively) were removed before fitting the conditional FDR models. The final models were constructed based on 500 iterations of randomly LD-pruned (r^2^ < 0.2) SNPs.

### Identification of independent genomic loci

Independent loci for each cFDR-GWAS were identified using FUMA^140^ v1.6.1 with default settings. In summary, independent lead SNPs were first identified based on LD estimated within the European samples from the 1000 Genomes Project Phase 3^138^ dataset using a cutoff of r^2^ = 0.1. Next, independent loci were obtained by merging any lead SNPs within 250 kb into the same locus represented by the most significant lead SNP. Any reference to “lead SNP” in the rest of the manuscript refers to only the most significant lead SNP at each independent locus. The final set of independent loci across all three cFDR-GWASs was obtained by merging any lead SNPs within 250 kb into the same locus represented by the most significant lead SNP.

### Estimation of the number of expected false discoveries

The expected number of false discoveries at a certain cFDR threshold was calculated as the sum of cFDR-values lower than the threshold among the independent lead SNPs of each cFDR-GWAS separately. This is possible since the conditional FDR is defined as a (posterior) probability and can therefore be treated as such. Next, the resulting three numbers were summed to yield a total number of expected false discoveries across all three cFDR-GWASs conducted. This estimate is conservative, given the high degree of dependence between the three cFDR-GWASs, meaning that the true number of false discoveries is lower.

### Gene annotation and overrepresentation analysis

Overrepresentation analysis of GO biological processes and mouse phenotypes among a set of lead SNPs was performed with GREAT^75^ v4.0.4 using default settings and a binomial test. Terms were considered significantly overrepresented among the set of lead SNPs at a 5% FDR. For each locus, up to two genes were annotated: the gene with the nearest transcription start site (TSS) centromeric from the lead SNP and the gene with the nearest TSS telomeric from the lead SNP on the condition that their TSSs were within 1 Mb of the lead SNP or within a curated regulatory region. For loci where none of the annotated genes were strongly supported by literature, a gene was manually annotated if it was within 1 Mb, had strong literature support (e.g., involved in craniofacial syndromes or with experimental evidence of involvement in craniofacial development), and was located in the same topologically associated domain based on micro-C in human embryonic stem cells^141^.

### REVIGO analysis

The list of GO biological processes used for REVIGO^76^ analysis was compiled by taking the union set of terms that were overrepresented (5% FDR) using the binomial test in GREAT among the loci (1% cFDR) from each cFDR-GWAS separately. A semantic space was then generated using REVIGO^76^ v1.8.1 with the SimRel^142^ similarity measure, which was the default option, and the list size set to “Large (0.9)”. Coordinates of terms in the obtained space are available from Supplementary Data 7.

For visualization purposes, terms were broadly categorized in three groups and “other”. Any term that contained “regulation” was assigned to the “regulation” category. Other terms that contained either “signal” or “response” were assigned to the “signaling group” and those that contained either “development”, “differentiation”, “formation”, or “-ogenesis” were assigned to the “development / morphogenesis / differentiation” group. All other terms were considered “other”. The final plots were made in Matlab 2023a.

### STRING analysis

Gene-interaction networks were constructed using STRING^143^ v12.0 using all interaction sources combined (text mining, experiments, databases, co-expression, neighborhood, gene fusion, co-occurrence; default) and a medium confidence cutoff (0.4; default). For added flexibility in visualizing the network, the final plots were made in Matlab 2023a based on the node coordinates and edge weights from the STRING exports.

### Stratified LD-score regression

GWAS summary statistics for the cranial vault shape GWAS in the European subset (*n* = 4198) of the ABCD cohort were obtained from our previous work^6^. LD scores were created for each annotation (corresponding to a set of differential or control distal ATAC-seq peaks) using the 1000 Genomes Phase 3 population reference^138^. Each annotation’s heritability enrichment was computed by adding the annotation to the baseline LD model and regressing against trait chi-squared statistics using HapMap3 SNPs with the S-LDSC^135^ package v.1.0.1. TWIST1-dependent ATAC-seq peaks, as well as all ATAC-seq peaks in CNCCs, were obtained from Kim et al.^83^ We note that the TWIST1-dependent peak sets span 0.67% and 0.73% of SNPs for acute depletion and long-term loss, respectively (based on 1000 Genomes SNP annotation in individuals of European ancestry, which encompass our GWAS populations), above the 0.5% defined as a large annotation.

### LocusZoom plots

Summary statistics of relevant GWASs^6,7,33^ were obtained from GWAS Catalog (GCST90270327; GCST90012880; and GCST90007266). LD with the lead SNP was obtained using the NIH LDlink^144^ (“LD proxy”) rest API, based on the 1000 Genomes Phase 3 GRCh38 High Coverage EUR genomes^138^. Protein coding genes including their exons were annotated using NCBI RefSeq annotations (available at: http://hgdownload.soe.ucsc.edu/goldenPath/hg19/bigZips/genes/hg19.ncbiRefSeq.gtf.gz). Plots were made in Matlab 2023b.

### Reporting summary

Further information on research design is available in the Nature Portfolio Reporting Summary linked to this article.

## Supplementary information

Supplementary Information

Description of Additional Supplementary Files

Supplementary Data 1–7

Supplementary Data 8

Reporting Summary

Transparent Peer Review file

## Data Availability

All the data and detailed information for the ABCD Study, including MRI scans, genetic markers, and covariates are available under restricted access through the ABCD data repository (https://nda.nih.gov/abcd/) upon completion of the relevant data use agreements. The ABCD data repository grows and changes over time and the data used in this work came from data release 3.0 (10.15154/1519007 and 10.15154/wthp-7h18). The NYGC 30 × 1000 genomes phased dataset and HGDP dataset are freely available online (http://ftp.1000genomes.ebi.ac.uk/vol1/ftp/data_collections/1000G_2504_high_coverage/working/20201028_3202_phased/, and https://ftp.sra.ebi.ac.uk/1000g/ftp/data_collections/HGDP/data/). The LD block coordinates used in this study are available from Berisa et al.^137^ at (https://bitbucket.org/nygcresearch/ldetect-data/src/master/). Mesh templates used for surface registration are available from the FigShare repositories of previous works (10.6084/m9.figshare.c.6858271.v1^145^, 10.6084/m9.figshare.7649024.v1^146^, and 10.6084/m9.figshare.c.5089841.v1^147^). Summary statistics for the cFDR-GWASs are available from FigShare (10.6084/m9.figshare.c.7680035.v1^148^). An overview of PubMed IDs and URLs for the GWAS summary statistics used in this work are provided in Supplementary Table 1. Transcription factor binding site coordinates can be obtained from the TF Link database v1.0 (https://tflink.net/download/, and https://cdn.netbiol.org/tflink/download_files/TFLink_Homo_sapiens_bindingSites_All_annotation_v1.0.tsv.gz). A list of genomic regions differentially accessible upon TWIST1 loss or depletion can be obtained from Kim et al.^83^ (GEO: GSE230319). RefSeq gene and exon annotations used in LocusZoom plots are freely available from the UCSC golden path (http://hgdownload.soe.ucsc.edu/goldenPath/hg19/bigZips/genes/hg19.ncbiRefSeq.gtf.gz). The Source data behind the graphs in the paper can be found in Supplementary Data 1–8.

## Code availability

KU Leuven provides the MeshMonk v.0.0.6 spatially dense facial-mapping software, free to use for academic purposes available at (https://github.com/TheWebMonks/meshmonk). The latest version is available from the FigShare repository of a previous publication (10.6084/m9.figshare.c.6858271.v1^145^). Matlab implementations of the hierarchical spectral clustering to obtain facial segmentations are available from a previous publication (10.6084/m9.figshare.7649024.v1^146^). The conditional FDR software is available from Github (https://github.com/precimed/pleiofdr). The statistical analyses in this work were based on functions in Matlab 2021a–2023b, python v3.7.6, R v4.2.1, PLINK 2.0, bcftools v1.10.2, vcftools v0.1.17, SHAPEIT v4.2.2, IMPUTE5 v1.1.5, imp5Chunker v1.1.5, ADMIXTURE v1.3.0, MeshMonk v0.0.6, GREAT v4.0.4, FUMA v1.6.1, SimpleITK v 2.1.0, REVIGO v1.8.1, FreeSurfer v6.0.0, StringDB v12.0, LDSC v1.0.1

## References

[1] Richtsmeier, J. T. & Flaherty, K. Hand in glove: brain and skull in development and dysmorphogenesis. *Acta Neuropathol. (Berl.)* **125**, 469–489 (2013). 23525521 10.1007/s00401-013-1104-y PMC3652528

[2] Lesciotto, K. M. & Richtsmeier, J. T. Craniofacial skeletal response to encephalization: How do we know what we think we know? *Am. J. Phys. Anthropol.* **168**, 27–46 (2019). 30680710 10.1002/ajpa.23766 PMC6424107

[3] Gokhman, D. et al. Human–chimpanzee fused cells reveal cis-regulatory divergence underlying skeletal evolution. *Nat. Genet.* **53**, 467–476 (2021). 33731941 10.1038/s41588-021-00804-3 PMC8038968

[4] Prescott, S. L. et al. Enhancer divergence and cis-regulatory evolution in the human and chimp neural crest. *Cell* **163**, 68–83 (2015). 26365491 10.1016/j.cell.2015.08.036 PMC4848043

[5] Rada-Iglesias, A., Prescott, S. L. & Wysocka, J. Human genetic variation within neural crest enhancers: Molecular and phenotypic implications. *Philos. Trans. R. Soc. B Biol. Sci.* **368**, 20120360 (2013). 10.1098/rstb.2012.0360 PMC3682725 23650634

[6] Goovaerts, S. et al. Joint multi-ancestry and admixed GWAS reveals the complex genetics behind human cranial vault shape. *Nat. Commun.* **14**, 7436 (2023). 37973980 10.1038/s41467-023-43237-8 PMC10654897

[7] White, J. D. et al. Insights into the genetic architecture of the human face. *Nat. Genet.* **53**, 45–53 (2021). 33288918 10.1038/s41588-020-00741-7 PMC7796995

[8] Stiles, J. & Jernigan, T. L. The basics of brain development. *Neuropsychol. Rev.* **20**, 327–348 (2010). 21042938 10.1007/s11065-010-9148-4 PMC2989000

[9] Minoux, M. & Rijli, F. M. Molecular mechanisms of cranial neural crest cell migration and patterning in craniofacial development. *Development* **137**, 2605–2621 (2010). 20663816 10.1242/dev.040048

[10] Santagati, F. & Rijli, F. M. Cranial neural crest and the building of the vertebrate head. *Nat. Rev. Neurosci.* **4**, 806–818 (2003). 14523380 10.1038/nrn1221

[11] Marcucio, R. S., Young, N. M., Hu, D. & Hallgrimsson, B. Mechanisms that underlie co-variation of the brain and face. *genesis* **49**, 177–189 (2011). 21381182 10.1002/dvg.20710 PMC3086711

[12] White, H. E., Goswami, A. & Tucker, A. S. The intertwined evolution and development of sutures and cranial morphology. *Front. Cell Dev. Biol.* **9**, 653579 (2021). 33842480 10.3389/fcell.2021.653579 PMC8033035

[13] Szabo-Rogers, H. L., Smithers, L. E., Yakob, W. & Liu, K. J. New directions in craniofacial morphogenesis. *Dev. Biol.* **341**, 84–94 (2010). 19941846 10.1016/j.ydbio.2009.11.021

[14] Twigg, S. R. F. & Wilkie, A. O. M. New insights into craniofacial malformations. *Hum. Mol. Genet.* **24**, R50–R59 (2015). 26085576 10.1093/hmg/ddv228 PMC4571997

[15] Inoue, T., Ota, M., Mikoshiba, K. & Aruga, J. *Zic2* and *Zic3* synergistically control neurulation and segmentation of paraxial mesoderm in mouse embryo. *Dev. Biol.* **306**, 669–684 (2007). 17490632 10.1016/j.ydbio.2007.04.003

[16] Nagai, T. et al. Zic2 regulates the kinetics of neurulation. *Proc. Natl. Acad. Sci.* **97**, 1618–1623 (2000). 10677508 10.1073/pnas.97.4.1618 PMC26484

[17] Stolt, C. C. et al. The Sox9 transcription factor determines glial fate choice in the developing spinal cord. *Genes Dev.* **17**, 1677–1689 (2003). 12842915 10.1101/gad.259003 PMC196138

[18] Selleri, L. & Rijli, F. M. Shaping faces: genetic and epigenetic control of craniofacial morphogenesis. *Nat. Rev. Genet.* **24**, 610–626 (2023). 37095271 10.1038/s41576-023-00594-w

[19] Flaherty, K., Singh, N. & Richtsmeier, J. T. Understanding craniosynostosis as a growth disorder. *WIREs Dev. Biol.* **5**, 429–459 (2016). 10.1002/wdev.227 PMC4911263 27002187

[20] Abdellaoui, A., Yengo, L., Verweij, K. J. H. & Visscher, P. M. 15 years of GWAS discovery: Realizing the promise. *Am. J. Hum. Genet.* **110**, 179–194 (2023). 36634672 10.1016/j.ajhg.2022.12.011 PMC9943775

[21] Sparks, C. S. & Jantz, R. L. A reassessment of human cranial plasticity: Boas revisited. *Proc. Natl. Acad. Sci.* **99**, 14636–14639 (2002). 12374854 10.1073/pnas.222389599 PMC137471

[22] Roosenboom, J. et al. Mapping genetic variants for cranial vault shape in humans. *PLoS ONE* **13**, e0196148 (2018). 29698431 10.1371/journal.pone.0196148 PMC5919379

[23] Coussens, A. K. et al. Linkage disequilibrium analysis identifies an FGFR1 haplotype-tag SNP associated with normal variation in craniofacial shape. *Genomics* **85**, 563–573 (2005). 15820308 10.1016/j.ygeno.2005.02.002

[24] Gómez-Valdés, J. A. et al. Fibroblast growth factor receptor 1 (FGFR1) variants and craniofacial variation in Amerindians and related populations. *Am. J. Hum. Biol.* **25**, 12–19 (2013). 23070782 10.1002/ajhb.22331

[25] Taal, H. R. et al. Common variants at 12q15 and 12q24 are associated with infant head circumference. *Nat. Genet.* **44**, 532–538 (2012). 22504419 10.1038/ng.2238 PMC3773913

[26] Haworth, S. et al. Low-frequency variation in TP53 has large effects on head circumference and intracranial volume. *Nat. Commun.* **10**, 357 (2019). 30664637 10.1038/s41467-018-07863-x PMC6341110

[27] Yang, X.-L. et al. Three novel loci for infant head circumference identified by a joint association analysis. *Front. Genet.* **10**, 947 (2019). 31681408 10.3389/fgene.2019.00947 PMC6798153

[28] Qin, X., Jiang, Q., Miyazaki, T. & Komori, T. Runx2 regulates cranial suture closure by inducing hedgehog, Fgf, Wnt and Pthlh signaling pathway gene expressions in suture mesenchymal cells. *Hum. Mol. Genet.* **28**, 896–911 (2019). 30445456 10.1093/hmg/ddy386

[29] Komori, T. Molecular mechanism of Runx2-dependent bone development. *Mol. Cells* **43**, 168–175 (2020). 31896233 10.14348/molcells.2019.0244 PMC7057844

[30] Komori, T. Regulation of proliferation, differentiation and functions of osteoblasts by Runx2. *Int. J. Mol. Sci.* **20**, 1694 (2019). 30987410 10.3390/ijms20071694 PMC6480215

[31] Komori, T. Whole aspect of Runx2 functions in skeletal development. *Int. J. Mol. Sci.* **23**, 5776 (2022). 35628587 10.3390/ijms23105776 PMC9144571

[32] Kawane, T. et al. Runx2 is required for the proliferation of osteoblast progenitors and induces proliferation by regulating Fgfr2 and Fgfr3. *Sci. Rep.* **8**, 13551 (2018). 30202094 10.1038/s41598-018-31853-0 PMC6131145

[33] Naqvi, S. et al. Shared heritability of human face and brain shape. *Nat. Genet.* **53**, 830–839 (2021). 33821002 10.1038/s41588-021-00827-w PMC8232039

[34] Claes, P. et al. Genome-wide mapping of global-to-local genetic effects on human facial shape. *Nat. Genet.* **50**, 414–423 (2018). 29459680 10.1038/s41588-018-0057-4 PMC5937280

[35] Bonfante, B. et al. A GWAS in Latin Americans identifies novel face shape loci, implicating VPS13B and a Denisovan introgressed region in facial variation. *Sci. Adv.* **7**, eabc6160 (2021). 33547071 10.1126/sciadv.abc6160 PMC7864580

[36] Shaffer, J. R. et al. Genome-wide association study reveals multiple loci influencing normal human facial morphology. *PLOS Genet* **12**, e1006149 (2016). 27560520 10.1371/journal.pgen.1006149 PMC4999139

[37] Zhang, M. et al. Genetic variants underlying differences in facial morphology in East Asian and European populations. *Nat. Genet.* **54**, 403–411 (2022). 35393595 10.1038/s41588-022-01038-7

[38] Hoskens, H. et al. 3D facial phenotyping by biometric sibling matching used in contemporary genomic methodologies. *PLOS Genet* **17**, e1009528 (2021). 33983923 10.1371/journal.pgen.1009528 PMC8118281

[39] Xiong, Z. et al. Combining genome-wide association studies highlight novel loci involved in human facial variation. *Nat. Commun.* **13**, 7832 (2022). 36539420 10.1038/s41467-022-35328-9 PMC9767941

[40] Li, Q. et al. Automatic landmarking identifies new loci associated with face morphology and implicates Neanderthal introgression in human nasal shape. *Commun. Biol.* **6**, 1–13 (2023). 37156940 10.1038/s42003-023-04838-7 PMC10167347

[41] Knol, M. J. et al. Genetic architecture of orbital telorism. *Hum. Mol. Genet.* **31**, 1531–1543 (2021). 10.1093/hmg/ddab334 PMC9071440 34791242

[42] Xiong, Z. et al. Novel genetic loci affecting facial shape variation in humans. *eLife* **8**, e49898 (2019). 31763980 10.7554/eLife.49898 PMC6905649

[43] Adhikari, K. et al. A genome-wide association scan implicates DCHS2, RUNX2, GLI3, PAX1 and EDAR in human facial variation. *Nat. Commun.* **7**, 11616 (2016). 27193062 10.1038/ncomms11616 PMC4874031

[44] Grasby, K. L. et al. The genetic architecture of the human cerebral cortex. *Science* **367**, eaay6690 (2020). 32193296 10.1126/science.aay6690 PMC7295264

[45] Hofer, E. et al. Genetic correlations and genome-wide associations of cortical structure in general population samples of 22,824 adults. *Nat. Commun.* **11**, 4796 (2020). 32963231 10.1038/s41467-020-18367-y PMC7508833

[46] Zhao, B. et al. Genome-wide association analysis of 19,629 individuals identifies variants influencing regional brain volumes and refines their genetic co-architecture with cognitive and mental health traits. *Nat. Genet.* **51**, 1637–1644 (2019). 31676860 10.1038/s41588-019-0516-6 PMC6858580

[47] Fan, C. C. et al. Multivariate genome-wide association study on tissue-sensitive diffusion metrics highlights pathways that shape the human brain. *Nat. Commun.* **13**, 2423 (2022). 35505052 10.1038/s41467-022-30110-3 PMC9065144

[48] van der Meer, D. et al. Understanding the genetic determinants of the brain with MOSTest. *Nat. Commun.* **11**, 3512 (2020). 32665545 10.1038/s41467-020-17368-1 PMC7360598

[49] Hibar, D. P. et al. Common genetic variants influence human subcortical brain structures. *Nature* **520**, 224–229 (2015). 25607358 10.1038/nature14101 PMC4393366

[50] van der Lee, S. J. et al. A genome-wide association study identifies genetic loci associated with specific lobar brain volumes. *Commun. Biol.* **2**, 1–9 (2019). 31396565 10.1038/s42003-019-0537-9 PMC6677735

[51] Zhao, B. et al. Large-scale GWAS reveals genetic architecture of brain white matter microstructure and genetic overlap with cognitive and mental health traits (n = 17,706). *Mol. Psychiatry* **26**, 3943–3955 (2021). 31666681 10.1038/s41380-019-0569-z PMC7190426

[52] Kague, E., Medina-Gomez, C., Boyadjiev, S. A. & Rivadeneira, F. The genetic overlap between osteoporosis and craniosynostosis. *Front. Endocrinol*. **13**, 1020821 (2022). 10.3389/fendo.2022.1020821 PMC9548872 36225206

[53] Medina-Gomez, C. et al. Bone mineral density loci specific to the skull portray potential pleiotropic effects on craniosynostosis. *Commun. Biol.* **6**, 1–12 (2023). 37402774 10.1038/s42003-023-04869-0 PMC10319806

[54] Andreassen, O. A. et al. Improved detection of common variants associated with schizophrenia and bipolar disorder using pleiotropy-informed conditional false discovery rate. *PLOS Genet* **9**, e1003455 (2013). 23637625 10.1371/journal.pgen.1003455 PMC3636100

[55] Hu, Y. et al. Identification of novel potentially pleiotropic variants associated with osteoporosis and obesity using the cFDR method. *J. Clin. Endocrinol. Metab.* **103**, 125–138 (2018). 29145611 10.1210/jc.2017-01531 PMC6061219

[56] Andreassen, O. A. et al. Identifying common genetic variants in blood pressure due to polygenic pleiotropy with associated phenotypes. *Hypertension* **63**, 819–826 (2014). 24396023 10.1161/HYPERTENSIONAHA.113.02077 PMC3984909

[57] Shadrin, A. A. et al. Novel loci associated with attention-deficit/hyperactivity disorder are revealed by leveraging polygenic overlap with educational attainment. *J. Am. Acad. Child Adolesc. Psychiatry* **57**, 86–95 (2018). 29413154 10.1016/j.jaac.2017.11.013 PMC5806128

[58] Tesfaye, M. et al. Shared genetic architecture between irritable bowel syndrome and psychiatric disorders reveals molecular pathways of the gut-brain axis. *Genome Med*. **15**, 60 (2023). 37528461 10.1186/s13073-023-01212-4 PMC10391890

[59] Lv, W.-Q. et al. Novel common variants associated with body mass index and coronary artery disease detected using a pleiotropic cFDR method. *J. Mol. Cell. Cardiol.* **112**, 1–7 (2017). 28843344 10.1016/j.yjmcc.2017.08.011 PMC5812278

[60] Smeland, O. B. et al. Discovery of shared genomic loci using the conditional false discovery rate approach. *Hum. Genet.* **139**, 85–94 (2020). 31520123 10.1007/s00439-019-02060-2

[61] Tissink, E. P. et al. Abundant pleiotropy across neuroimaging modalities identified through a multivariate genome-wide association study. *Nat. Commun.* **15**, 2655 (2024). 38531894 10.1038/s41467-024-46817-4 PMC10965919

[62] Goovaerts, S. et al. The impact of breastfeeding on facial appearance in adolescent children. *PLOS ONE* **19**, e0310538 (2024). 39288146 10.1371/journal.pone.0310538 PMC11407646

[63] Casey, B. J. et al. The adolescent brain cognitive development (ABCD) study: Imaging acquisition across 21 sites. *Dev. Cogn. Neurosci.* **32**, 43–54 (2018). 29567376 10.1016/j.dcn.2018.03.001 PMC5999559

[64] Hagler, D. J. et al. Image processing and analysis methods for the adolescent brain cognitive development study. *NeuroImage* **202**, 116091 (2019). 31415884 10.1016/j.neuroimage.2019.116091 PMC6981278

[65] Morris, J. A. et al. An atlas of genetic influences on osteoporosis in humans and mice. *Nat. Genet.* **51**, 258–266 (2019). 30598549 10.1038/s41588-018-0302-x PMC6358485

[66] Jansen, I. E. et al. Genome-wide meta-analysis identifies new loci and functional pathways influencing Alzheimer’s disease risk. *Nat. Genet.* **51**, 404–413 (2019). 30617256 10.1038/s41588-018-0311-9 PMC6836675

[67] Wuttke, M. et al. A catalog of genetic loci associated with kidney function from analyses of a million individuals. *Nat. Genet.* **51**, 957–972 (2019). 31152163 10.1038/s41588-019-0407-x PMC6698888

[68] Mbatchou, J. et al. Computationally efficient whole-genome regression for quantitative and binary traits. *Nat. Genet.* **53**, 1097–1103 (2021). 34017140 10.1038/s41588-021-00870-7

[69] Yengo, L. et al. A saturated map of common genetic variants associated with human height. *Nature* **610**, 704–712 (2022). 36224396 10.1038/s41586-022-05275-y PMC9605867

[70] de Lange, K. M. et al. Genome-wide association study implicates immune activation of multiple integrin genes in inflammatory bowel disease. *Nat. Genet.* **49**, 256–261 (2017). 28067908 10.1038/ng.3760 PMC5289481

[71] Koskeridis, F. et al. Pleiotropic genetic architecture and novel loci for C-reactive protein levels. *Nat. Commun.* **13**, 6939 (2022). 36376304 10.1038/s41467-022-34688-6 PMC9663411

[72] Vuckovic, D. et al. The polygenic and monogenic basis of blood traits and diseases. *Cell* **182**, 1214–1231.e11 (2020). 32888494 10.1016/j.cell.2020.08.008 PMC7482360

[73] Ripke, S. et al. Biological insights from 108 schizophrenia-associated genetic loci. *Nature* **511**, 421–427 (2014). 25056061 10.1038/nature13595 PMC4112379

[74] Efron, B. Size, power and false discovery rates. *Ann. Stat.* **35**, 1351–1377 (2007).

[75] McLean, C. Y. et al. GREAT improves functional interpretation of cis-regulatory regions. *Nat. Biotechnol.* **28**, 495–501 (2010). 20436461 10.1038/nbt.1630 PMC4840234

[76] Supek, F., Bošnjak, M., Škunca, N. & Šmuc, T. REVIGO summarizes and visualizes long lists of gene ontology terms. *PLOS ONE* **6**, e21800 (2011). 21789182 10.1371/journal.pone.0021800 PMC3138752

[77] Hammond, R. K. et al. Biological constraints on GWAS SNPs at suggestive significance thresholds reveal additional BMI loci. *eLife* **10**, e62206 (2021). 33459256 10.7554/eLife.62206 PMC7815306

[78] Mizuguchi, T. et al. Loss-of-function and gain-of-function mutations in PPP3CA cause two distinct disorders. *Hum. Mol. Genet.* **27**, 1421–1433 (2018). 29432562 10.1093/hmg/ddy052

[79] Arboleda, V. A. et al. Mutations in the PCNA-binding domain of CDKN1C cause IMAGe syndrome. *Nat. Genet.* **44**, 788–792 (2012). 22634751 10.1038/ng.2275 PMC3386373

[80] Topa, A. et al. The value of genome-wide analysis in craniosynostosis. *Front. Genet*. **14**, 1322462 (2024). 10.3389/fgene.2023.1322462 PMC10839781 38318288

[81] Twigg, S. R. F. & Wilkie, A. O. M. A genetic-pathophysiological framework for craniosynostosis. *Am. J. Hum. Genet.* **97**, 359–377 (2015). 26340332 10.1016/j.ajhg.2015.07.006 PMC4564941

[82] Liska, O. et al. TFLink: an integrated gateway to access transcription factor–target gene interactions for multiple species. *Database* **2022**, baac083 (2022). 36124642 10.1093/database/baac083 PMC9480832

[83] Kim, S. et al. DNA-guided transcription factor cooperativity shapes face and limb mesenchyme. *Cell* **187**, 692–711.e26 (2024). 38262408 10.1016/j.cell.2023.12.032 PMC10872279

[84] Korvala, J. et al. Mutations in LRP5 cause primary osteoporosis without features of OI by reducing Wnt signaling activity. *BMC Med. Genet.* **13**, 26 (2012). 22487062 10.1186/1471-2350-13-26 PMC3374890

[85] Kwee, M. L. et al. An autosomal dominant high bone mass phenotype in association with craniosynostosis in an extended family is caused by an LRP5 missense mutation. *J. Bone Miner. Res.* **20**, 1254–1260 (2005). 15940380 10.1359/JBMR.050303

[86] Han, J. et al. Concerted action of *Msx1* and *Msx2* in regulating cranial neural crest cell differentiation during frontal bone development. *Mech. Dev.* **124**, 729–745 (2007). 17693062 10.1016/j.mod.2007.06.006 PMC2220014

[87] Liang, J. et al. MSX1 mutations and associated disease phenotypes: genotype-phenotype relations. *Eur. J. Hum. Genet.* **24**, 1663–1670 (2016). 27381090 10.1038/ejhg.2016.78 PMC5117928

[88] Jin, S.-W., Sim, K.-B. & Kim, S.-D. Development and growth of the normal cranial vault: An embryologic review. *J. Korean Neurosurg. Soc.* **59**, 192–196 (2016). 27226848 10.3340/jkns.2016.59.3.192 PMC4877539

[89] Solovieff, N., Cotsapas, C., Lee, P. H., Purcell, S. M. & Smoller, J. W. Pleiotropy in complex traits: Challenges and strategies. *Nat. Rev. Genet.* **14**, 483–495 (2013). 23752797 10.1038/nrg3461 PMC4104202

[90] Gaur, T. et al. Canonical WNT signaling promotes osteogenesis by directly stimulating *Runx2* gene expression. *J. Biol. Chem.* **280**, 33132–33140 (2005). 16043491 10.1074/jbc.M500608200

[91] Al‐Bari, A. A. & Al Mamun, A. Current advances in regulation of bone homeostasis. *FASEB BioAdv*. **2**, 668–679 (2020). 33205007 10.1096/fba.2020-00058 PMC7655096

[92] Guo, Y. et al. BMP-IHH-mediated interplay between mesenchymal stem cells and osteoclasts supports calvarial bone homeostasis and repair. *Bone Res*. **6**, 30 (2018). 10.1038/s41413-018-0031-x PMC6193039 30345151

[93] Ishii, M. et al. Msx2 and Twist cooperatively control the development of the neural crest-derived skeletogenic mesenchyme of the murine skull vault. *Development* **130**, 6131–6142 (2003). 14597577 10.1242/dev.00793

[94] Nakashima, K. et al. The novel zinc finger-containing transcription factor osterix is required for osteoblast differentiation and bone formation. *Cell* **108**, 17–29 (2002). 11792318 10.1016/s0092-8674(01)00622-5

[95] Pan, A., Chang, L., Nguyen, A. & James, A. W. A review of hedgehog signaling in cranial bone development. *Front. Physiol.* **4**, 61 (2013). 23565096 10.3389/fphys.2013.00061 PMC3613593

[96] Warren, S. M., Brunet, L. J., Harland, R. M., Economides, A. N. & Longaker, M. T. The BMP antagonist noggin regulates cranial suture fusion. *Nature* **422**, 625–629 (2003). 12687003 10.1038/nature01545

[97] Matsubara, T. et al. BMP2 regulates osterix through Msx2 and Runx2 during osteoblast differentiation∗. *J. Biol. Chem.* **283**, 29119–29125 (2008). 18703512 10.1074/jbc.M801774200 PMC2662012

[98] Lee, M.-H. et al. Dlx5 specifically regulates Runx2 type II expression by binding to homeodomain-response elements in the Runx2 distal promoter*. *J. Biol. Chem.* **280**, 35579–35587 (2005). 16115867 10.1074/jbc.M502267200

[99] Gadi, J. et al. The transcription factor protein Sox11 enhances early osteoblast differentiation by facilitating proliferation and the survival of mesenchymal and osteoblast progenitors. *J. Biol. Chem.* **288**, 25400–25413 (2013). 23888050 10.1074/jbc.M112.413377 PMC3757203

[100] Boulet, S. L., Rasmussen, S. A. & Honein, M. A. A population-based study of craniosynostosis in metropolitan Atlanta, 1989–2003. *Am. J. Med. Genet. A.* **146A**, 984–991 (2008). 18344207 10.1002/ajmg.a.32208

[101] Ma, L., Golden, S., Wu, L. & Maxson, R. The molecular basis of Boston-type craniosynostosis: the Pro148–>His mutation in the N-terminal arm of the MSX2 homeodomain stabilizes DNA binding without altering nucleotide sequence preferences. *Hum. Mol. Genet.* **5**, 1915–1920 (1996). 8968743 10.1093/hmg/5.12.1915

[102] Plaisancié, J. et al. MSX2 gene duplication in a patient with eye development defects. *Ophthalmic Genet* **36**, 353–358 (2015). 24666290 10.3109/13816810.2014.886270

[103] Timberlake, A. T. et al. De novo mutations in the BMP signaling pathway in lambdoid craniosynostosis. *Hum. Genet.* **142**, 21–32 (2023). 35997807 10.1007/s00439-022-02477-2

[104] Klopocki, E. et al. Copy-number variations involving the IHH locus are associated with syndactyly and craniosynostosis. *Am. J. Hum. Genet.* **88**, 70–75 (2011). 21167467 10.1016/j.ajhg.2010.11.006 PMC3014361

[105] Varvagiannis, K. et al. Pure de novo partial trisomy 6p in a girl with craniosynostosis. *Am. J. Med. Genet. A.* **161A**, 343–351 (2013). 23307468 10.1002/ajmg.a.35727

[106] Mefford, H. C. et al. Copy number variation analysis in single-suture craniosynostosis: Multiple rare variants including RUNX2 duplication in two cousins with metopic craniosynostosis. *Am. J. Med. Genet. A.* **152A**, 2203–2210 (2010). 20683987 10.1002/ajmg.a.33557 PMC3104131

[107] Wilkie, A. O. M. et al. Functional haploinsufficiency of the human homeobox gene MSX2 causes defects in skull ossification. *Nat. Genet.* **24**, 387–390 (2000). 10742103 10.1038/74224

[108] Mundlos, S. Cleidocranial dysplasia: clinical and molecular genetics. *J. Med. Genet.* **36**, 177–182 (1999). 10204840 PMC1734317

[109] El Ghouzzi, V. et al. Mutations within or upstream of the basic helix-loop-helix domain of the TWIST gene are specific to Saethre-Chotzen syndrome. *Eur. J. Hum. Genet. EJHG* **7**, 27–33 (1999). 10094188 10.1038/sj.ejhg.5200240

[110] Gripp, K. W., Zackai, E. H. & Stolle, C. A. Mutations in the human TWIST gene. *Hum. Mutat.* **15**, 150–155 (2000). 10649491 10.1002/(SICI)1098-1004(200002)15:2<150::AID-HUMU3>3.0.CO;2-D

[111] He (何璇), X. A. et al. Identification of conserved skeletal enhancers associated with craniosynostosis risk genes. *Hum. Mol. Genet*. ddad182 10.1093/hmg/ddad182 (2023). 10.1093/hmg/ddad182 PMC11070136 37883470

[112] Justice, C. M. et al. A genome-wide association study implicates the BMP7 locus as a risk factor for nonsyndromic metopic craniosynostosis. *Hum. Genet.* **139**, 1077–1090 (2020). 32266521 10.1007/s00439-020-02157-z PMC7415527

[113] Cm, J. et al. A genome-wide association study identifies susceptibility loci for nonsyndromic sagittal craniosynostosis near BMP2 and within BBS9. *Nat. Genet*. **44**, 1360–1364 (2012). 10.1038/ng.2463 PMC3736322 23160099

[114] Musolf, A. M. et al. Whole genome sequencing identifies associations for nonsyndromic sagittal craniosynostosis with the intergenic region of BMP2 and noncoding RNA gene LINC01428. *Sci. Rep.* **14**, 8533 (2024). 38609424 10.1038/s41598-024-58343-w PMC11014861

[115] Nicoletti, P. et al. Regulatory elements in *SEM1–DLX5–DLX6* (7q21.3) locus contribute to genetic control of coronal nonsyndromic craniosynostosis and bone density-related traits. *Genet. Med. Open* 101851 10.1016/j.gimo.2024.101851 (2024). 10.1016/j.gimo.2024.101851 PMC11434253 39345948

[116] Pepper, J., Bhattacharyya, S. & Gallo, P. Undiagnosed sagittal synostosis as cause of “idiopathic” intracranial hypertension. *Childs Nerv. Syst*. 10.1007/s00381-024-06308-9 (2024). 10.1007/s00381-024-06308-9 38353695

[117] Manrique, M. et al. Normocephalic sagittal craniosynostosis in young children is common and unrecognized. *Childs Nerv. Syst.* **38**, 1549–1556 (2022). 35716184 10.1007/s00381-022-05533-4

[118] Dudakovic, A., Nam, H. K., van Wijnen, A. J. & Hatch, N. E. Genetic background dependent modifiers of craniosynostosis severity. *J. Struct. Biol.* **212**, 107629 (2020). 32976998 10.1016/j.jsb.2020.107629 PMC7885185

[119] Niederreither, K. & Dollé, P. Retinoic acid in development: towards an integrated view. *Nat. Rev. Genet.* **9**, 541–553 (2008). 18542081 10.1038/nrg2340

[120] Sandell, L. L. et al. RDH10 is essential for synthesis of embryonic retinoic acid and is required for limb, craniofacial, and organ development. *Genes Dev.* **21**, 1113–1124 (2007). 17473173 10.1101/gad.1533407 PMC1855236

[121] James, A. W., Levi, B., Xu, Y., Carre, A. L. & Longaker, M. T. Retinoic acid enhances osteogenesis in cranial suture–derived mesenchymal cells: Potential mechanisms of retinoid-induced craniosynostosis. *Plast. Reconstr. Surg.* **125**, 1352 (2010). 20134361 10.1097/PRS.0b013e3181d62980 PMC2909493

[122] Roa, L. A., Bloemen, M., Carels, C. E. L., Wagener, F. A. D. T. G. & Von den Hoff, J. W. Retinoic acid disrupts osteogenesis in pre-osteoblasts by down-regulating WNT signaling. *Int. J. Biochem. Cell Biol.* **116**, 105597 (2019). 31479736 10.1016/j.biocel.2019.105597

[123] Abu-Abed, S. et al. The retinoic acid-metabolizing enzyme, CYP26A1, is essential for normal hindbrain patterning, vertebral identity, and development of posterior structures. *Genes Dev.* **15**, 226–240 (2001). 11157778 10.1101/gad.855001 PMC312609

[124] Laue, K. et al. Craniosynostosis and multiple skeletal anomalies in humans and zebrafish result from a defect in the localized degradation of retinoic acid. *Am. J. Hum. Genet.* **89**, 595–606 (2011). 22019272 10.1016/j.ajhg.2011.09.015 PMC3213388

[125] Grand, K. et al. Nonlethal presentations of CYP26B1-related skeletal anomalies and multiple synostoses syndrome. *Am. J. Med. Genet. A.* **185**, 2766–2775 (2021). 34160123 10.1002/ajmg.a.62387

[126] Silveira, K. C. et al. CYP26B1-related disorder: expanding the ends of the spectrum through clinical and molecular evidence. *Hum. Genet.* **142**, 1571–1586 (2023). 37755482 10.1007/s00439-023-02598-2 PMC10602971

[127] Quadro, L. et al. Pathways of vitamin A delivery to the embryo: Insights from a new tunable model of embryonic vitamin A deficiency. *Endocrinology* **146**, 4479–4490 (2005). 15994349 10.1210/en.2005-0158

[128] Alfaro-Almagro, F. et al. Image processing and Quality Control for the first 10,000 brain imaging datasets from UK Biobank. *Neuroimage* **166**, 400–424 (2018). 29079522 10.1016/j.neuroimage.2017.10.034 PMC5770339

[129] Turley, P. et al. Multi-trait analysis of genome-wide association summary statistics using MTAG. *Nat. Genet.* **50**, 229–237 (2018). 29292387 10.1038/s41588-017-0009-4 PMC5805593

[130] Campos, A. I. et al. Boosting the power of genome-wide association studies within and across ancestries by using polygenic scores. *Nat. Genet.* **55**, 1769–1776 (2023). 37723263 10.1038/s41588-023-01500-0

[131] Bennett, D., O’Shea, D., Ferguson, J., Morris, D. & Seoighe, C. Controlling for background genetic effects using polygenic scores improves the power of genome-wide association studies. *Sci. Rep.* **11**, 19571 (2021). 34599249 10.1038/s41598-021-99031-3 PMC8486788

[132] Dale, A. M., Fischl, B. & Sereno, M. I. Cortical surface-based analysis: I. Segmentation and surface reconstruction. *NeuroImage* **9**, 179–194 (1999). 9931268 10.1006/nimg.1998.0395

[133] White, J. D. et al. MeshMonk: Open-source large-scale intensive 3D phenotyping. *Sci. Rep.* **9**, 6085 (2019). 30988365 10.1038/s41598-019-42533-y PMC6465282

[134] Manichaikul, A. et al. Robust relationship inference in genome-wide association studies. *Bioinformatics* **26**, 2867–2873 (2010). 20926424 10.1093/bioinformatics/btq559 PMC3025716

[135] Bulik-Sullivan, B. K. et al. LD Score regression distinguishes confounding from polygenicity in genome-wide association studies. *Nat. Genet.* **47**, 291–295 (2015). 25642630 10.1038/ng.3211 PMC4495769

[136] Altshuler, D. M. et al. Integrating common and rare genetic variation in diverse human populations. *Nature* **467**, 52–58 (2010). 20811451 10.1038/nature09298 PMC3173859

[137] Berisa, T. & Pickrell, J. K. Approximately independent linkage disequilibrium blocks in human populations. *Bioinformatics* **32**, 283–285 (2016). 26395773 10.1093/bioinformatics/btv546 PMC4731402

[138] Auton, A. et al. A global reference for human genetic variation. *Nature* **526**, 68–74 (2015). 26432245 10.1038/nature15393 PMC4750478

[139] van der Deen, M. et al. Genomic promoter occupancy of runt-related transcription factor RUNX2 in osteosarcoma cells identifies genes involved in cell adhesion and motility. *J. Biol. Chem.* **287**, 4503–4517 (2012). 22158627 10.1074/jbc.M111.287771 PMC3281617

[140] Watanabe, K., Taskesen, E., van Bochoven, A. & Posthuma, D. Functional mapping and annotation of genetic associations with FUMA. *Nat. Commun.* **8**, 1826 (2017). 29184056 10.1038/s41467-017-01261-5 PMC5705698

[141] Krietenstein, N. et al. Ultrastructural details of mammalian chromosome architecture. *Mol. Cell* **78**, 554–565.e7 (2020). 32213324 10.1016/j.molcel.2020.03.003 PMC7222625

[142] Schlicker, A., Domingues, F. S., Rahnenführer, J. & Lengauer, T. A new measure for functional similarity of gene products based on Gene Ontology. *BMC Bioinforma.* **7**, 302 (2006). 10.1186/1471-2105-7-302 PMC1559652 16776819

[143] Szklarczyk, D. et al. The STRING database in 2023: Protein–protein association networks and functional enrichment analyses for any sequenced genome of interest. *Nucleic Acids Res* **51**, D638–D646 (2023). 36370105 10.1093/nar/gkac1000 PMC9825434

[144] Machiela, M. J. & Chanock, S. J. LDlink: A web-based application for exploring population-specific haplotype structure and linking correlated alleles of possible functional variants. *Bioinformatics* **31**, 3555–3557 (2015). 26139635 10.1093/bioinformatics/btv402 PMC4626747

[145] Goovaerts, S. Joint multi-ancestry and admixed GWAS reveals the complex genetics behind human cranial vault shape. *FigShare*10.6084/m9.figshare.c.6858271.v1 (2023). 10.1038/s41467-023-43237-8 PMC10654897 37973980

[146] Sero, D. & Claes, P. Facial recognition from DNA using face-to-DNA-classifiers. *FigShare*10.6084/m9.figshare.7649024.v1 (2019). 10.1038/s41467-019-10617-y PMC6560034 31186421

[147] Naqvi, S. Shared heritability of face and brain shape. *FigShare*10.6084/m9.figshare.c.5089841.v1 (2021).

[148] Goovaerts, S. Enhanced insights into the genetic architecture of 3D cranial vault shape using pleiotropy-informed GWAS. *FigShare*10.6084/m9.figshare.c.7680035.v1 (2025). 10.1038/s42003-025-07875-6 PMC11909261 40087503

