# Supplementary material for: Enhanced insights into the genetic architecture of 3D cranial vault shape using pleiotropy-informed GWAS
Source: Commun Biol. 2025 Mar 15;8:439. doi: 10.1038/s42003-025-07875-6 (PMC11909261; doi:10.1038/s42003-025-07875-6)
Supplement: Supplementary file 1 — Supplementary Information [file 42003_2025_7875_MOESM1_ESM.pdf]

Supplementary Information for

Enhanced insights into the genetic architecture of 3D cranial vault shape using pleiotropy-informed GWAS

Seppe Goovaerts* *et al.*

*Corresponding author. Email: seppe.goovaerts@kuleuven.be

This PDF file includes:

Supplementary Note
Supplementary Fig. 1 and 2
Supplementary Table 1

Other Supplementary Files for this manuscript include the following:

Supplementary Data 1 to 8

Supplementary Note

Sample selection

The ABCD MRI data contain various imaging artefacts, such as compression of the craniofacial soft tissue due to subject fixation, earphones, and goggles, as well as uneven cranial vault surfaces caused by dense hair structures like braids. These artefacts can disproportionately affect individuals of different ancestries due to differences in hair types and facial structures. Prior to examining the morphological variance explained in the cranial vault by the brain and face, we selected an ancestrally homogeneous sample from the ABCD cohort to minimize these artefact-induced variabilities, which could otherwise lead to unpredictably biased results. Samples were selected based on their inferred ancestry proportions as described in detail in previous work[1]. Briefly, the combined reference set of individuals from the 1000 Genomes Project[2] and Human Diversity Project (HGDP)[3] was refined using unsupervised clustering (K = 6) in ADMIXTURE[4] followed by k-means (elkan algorithm from sklearn.cluster in Python v3.7.6) clustering on the obtained ancestry proportions, and keeping only samples with a higher-than-average main ancestry component across all samples from each resulting cluster. Subsequently, the refined set of reference individuals was used to supervise the clustering (K = 6) of the ABCD individuals in ADMIXTURE[4]. Samples with a >90% combined European, African, and American ancestry component were kept for further analysis, which included most of the ABCD cohort. A subset of European individuals from the ABCD cohort was obtained by k-means (elkan algorithm from sklearn.cluster in Python v3.7.6) clustering on the ancestry components inferred by the supervised ADMIXTURE[4] analysis, followed by the selection of individuals with a higher-than-average European ancestry component from the resulting European cluster.

Genotype imputation quality control

Genotypes were downloaded from the NIMH data archive. Imputation and genetic quality control has been described in detail in previous work[1]. Briefly, genotype files were converted to variant call format (VCF) using PLINK[5] 2.0 and dbSNP154 rsID's were annotated using Bcftools[6] (*annotate*). Phasing was performed on SNPs only ($n = 502{,}882$) using SHAPEIT[7] v4.2.2 with default parameter settings and the New York Genome Center (NYGC) 30x-1000 genomes-phased-dataset (http://ftp.1000genomes.ebi.ac.uk/vol1/ftp/data_collections/1000G_2504_high_coverage/working/20201028_3202_phased/) as reference. The reference dataset for imputation was compiled with data from the Human Genome Diversity Project[3] and the 1000 Genomes Project[2] (https://ftp.sra.ebi.ac.uk/1000g/ftp/data_collections/HGDP/data/) and prepared by the *Iliad*[8] genomic data pipeline. Imputation of ABCD genotypes was subsequently performed using imp5Chunker_v1.1.5[9] and IMPUTE5[9] v1.1.5 with SHAPEIT4 GRCh38 genetic maps and the combined NYGC1000G-HGDP reference dataset using default parameter settings. Genotypes were filtered for INFO scores > 0.3, minor allele frequency > 1%, and minimum genotyping rate of 95%. Since the conditional GWASs were run using summary statistics from prior works, any use of individual-level genotypes in this work was limited to the sets of conditional GWAS lead SNPs.

Image Acquisition, preprocessing, and quality control

Whole head 3T, minimally processed T1-weighted MR scans in NIFTI format were accessed through the NIMH data archive. Initial preprocessing steps included distortion correction, movement correction, resampling (1mm isotropic voxels), alignment to standard space, and initial quality control[10,11].

Cranial vault and facial surface extraction

Following our previous work[1], noise and imaging artefacts in the minimally processed MR images were reduced prior to any surface extraction. Briefly, for each image in the dataset this involved inter-subject non-rigid registrations of 300 other images from the dataset to that image using Elastix (SimpleITK library[12] in Python) and computing a consensus image based on the median intensity values per voxel. The registrations were performed with the *Param0000* parameter map (affine and B-spline)[13] using images that were matched in terms of sex at birth, height, weight, and genomic ancestry. Prior to extracting the face or cranial vault surface, the iso-surface of the full head was extracted (Matlab 2023a, *isosurface*), followed by removal of any internal structures based on the distance of each vertex to the centroid.

To extract the cranial vault, a mesh template of the full head surface (n = 28,218 vertices) was first rigidly and then non-rigidly registered onto the cleaned iso-surface from each participant using the MeshMonk[14] toolbox. The ears and surrounding region were ignored during the registration. Next, the cranial vault region (n = 11,410 vertices), which encompasses the supraorbital ridge and

extends towards the occipital bone was manually delineated on the template and subsequently cropped from each full head surface.

To extract the facial surface from the denoised MR images, a mesh template of the facial surface (n = 7160 vertices) was first rigidly and then non-rigidly registered onto the cleaned iso-surface from each participant using the MeshMonk[14] toolbox.

Cranial vault quality control

To identify outliers, a statistical shape model of cranial vault shape was built with PCA and by retaining PCs that capture 98% of the variance. Following previous work[1,15,16], the Mahalanobis distance from each cranial vault to the average cranial vault was transformed into a Z-score and any image with a Z-score larger than 1.5 was removed from further analysis.

Facial quality control

The facial surfaces obtained from MRI scans exhibited noise and artifacts caused by MR bias field, subject fixation, and partial volume effects. Nearly all facial surfaces were impacted by some degree of soft tissue compression, particularly in the cheeks or chin due to subject fixation, complicating the manual selection of high-quality images. To address this challenge, we devised a data-driven pipeline for quality control and sample selection to identify high-quality images more effectively.

To begin, we utilized noise-reduced T1-w MRI images to extract iso-surfaces (*isosurface,* Matlab 2023a). Subsequently, internal structures were removed based on vertex distance from the centroid

of the mesh, an optional step implemented to minimize potential surface registration errors later. Faces were then extracted from these iso-surfaces as mesh structures in dense correspondence using Meshmonk[14], involving both rigid and non-rigid surface registration with a facial mesh template (n = 7160 vertices). Imaging artifacts were identified in a vertex-wise manner based on a statistical shape model derived from a manually curated set of high-quality images (n = 4355) within the dataset.

To enhance the model's efficacy in detecting soft-tissue compression, we first mitigated this compression in the images used for constructing the reference model. This involved generating average facial surfaces for each MRI scanner and employing principal component analysis (PCA) to model machine-related shape variations, including soft-tissue compression. Any shape variation explained by the principal components accounting for 96% of the variation across the MRI averages was subtracted from the initially selected high-quality images. This process visually reduced the subtle soft-tissue compression that was still present in these images. Subsequently, a reference model was built from these "clean" images using PCA, which was then employed to detect imaging artifacts, such as soft-tissue compression, on a vertex-wise basis.

For each of the 7160 facial vertices, we established a distribution of 3D Euclidean distances between its position on the average face and those on the reference faces. This enabled us to evaluate each vertex on a new face against the reference model, producing a Z-score for each vertex that was converted into an outlier probability using a sigmoid activation function. A leave-one-out approach was employed to evaluate the high-quality images utilized in constructing the statistical

shape model. The resulting outlier maps (**Fig S2a**) illustrated the capability of this approach to automatically and scalably detect various types of imaging-related artifacts.

Sample selection was carried out utilizing the outlier maps generated through a K-means clustering methodology. Initially, a subset of 500 cleanest shapes was identified by evaluating the total number of vertices with an outlier probability below 0.05. Given the strong correlation among outlier probabilities across vertices, we employed PCA to reduce the dimensionality of the outlier maps, retaining only components that accounted for more than 1% of the variance. Subsequently, K-means++ clustering, with the number of clusters determined by the variance ratio criterion, was performed on the dimensionality-reduced outlier maps. Any images within clusters devoid of top 500 shapes were flagged for further review (**Fig S2b**). To ensure robustness, the clustering process was iterated 1000 times, and images flagged in at least 5% of the rounds were excluded from further analysis.

In order to enhance the resolution for artifact detection, we subdivided the face into smaller hierarchical modules through hierarchical spectral clustering, utilizing Escoufier's RV coefficient[17], following a similar approach as described in prior studies[15,16] (**Fig S2c**). Subsequently, we applied the K-means++ procedure to each resulting facial module (n = 31) as outlined previously. Ultimately, only faces that successfully passed the sample selection criteria within each of the 31 modules were retained for subsequent analysis (n = 4930).

<u>Cortical surface extraction and quality control</u>

Following previous work[18], the cortical surfaces of ABCD participants were segmented from T1-weighted MRIs using FreeSurfer's[19] v6.0.0 *recon-all* command and converted to the Connectivity

Informatics Technology Initiative file format (CIFTIFY), which is similar to the Human Connectome Project[20] (HCP) file format. From the CIFTIFY output, the mid-cortical surface was selected based on the Conte-69 atlas, for both the left and right hemisphere. After Procrustes superimposition of the 3D surface meshes (n = 29,759 vertices) representing the left and reflected right hemispheres, symmetrical shapes were obtained by taking the vertex-wise average of the superimposed hemispheres. Following PCA and retaining PCs that capture 80% of the variance, a Z-score was obtained as described for cranial vault shape, and all images with a Z-score > 3 were manually checked for artefacts.

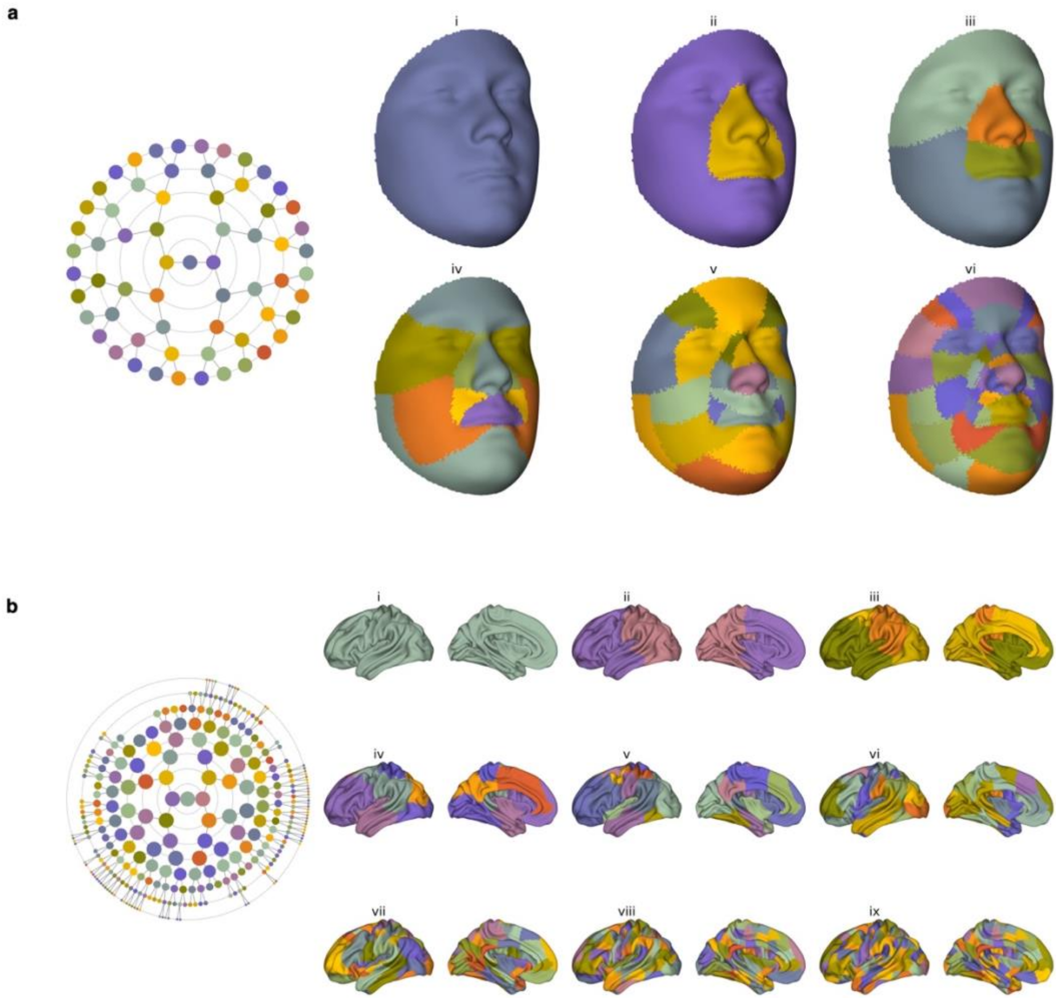

Supplementary Fig. 1. Hierarchical segmentation of the facial and mid-cortical surface. (a) Hierarchical segmentation of the facial surface into 63 segments across 6 levels (i to vi) as done in White et al.[15] **(b)** Hierarchical segmentation of the mid-cortical surface into 285 segments across 9 levels (i to ix), adapted from Naqvi et al.[18] Roman numerals in **(a)** and **(b)** indicate the hierarchical levels, with i indicating the full facial or cortical surface. Roman numerals also correspond to concentric circles in the respective rosettes, starting from the center. Each node corresponds to a single facial or cortical segment.

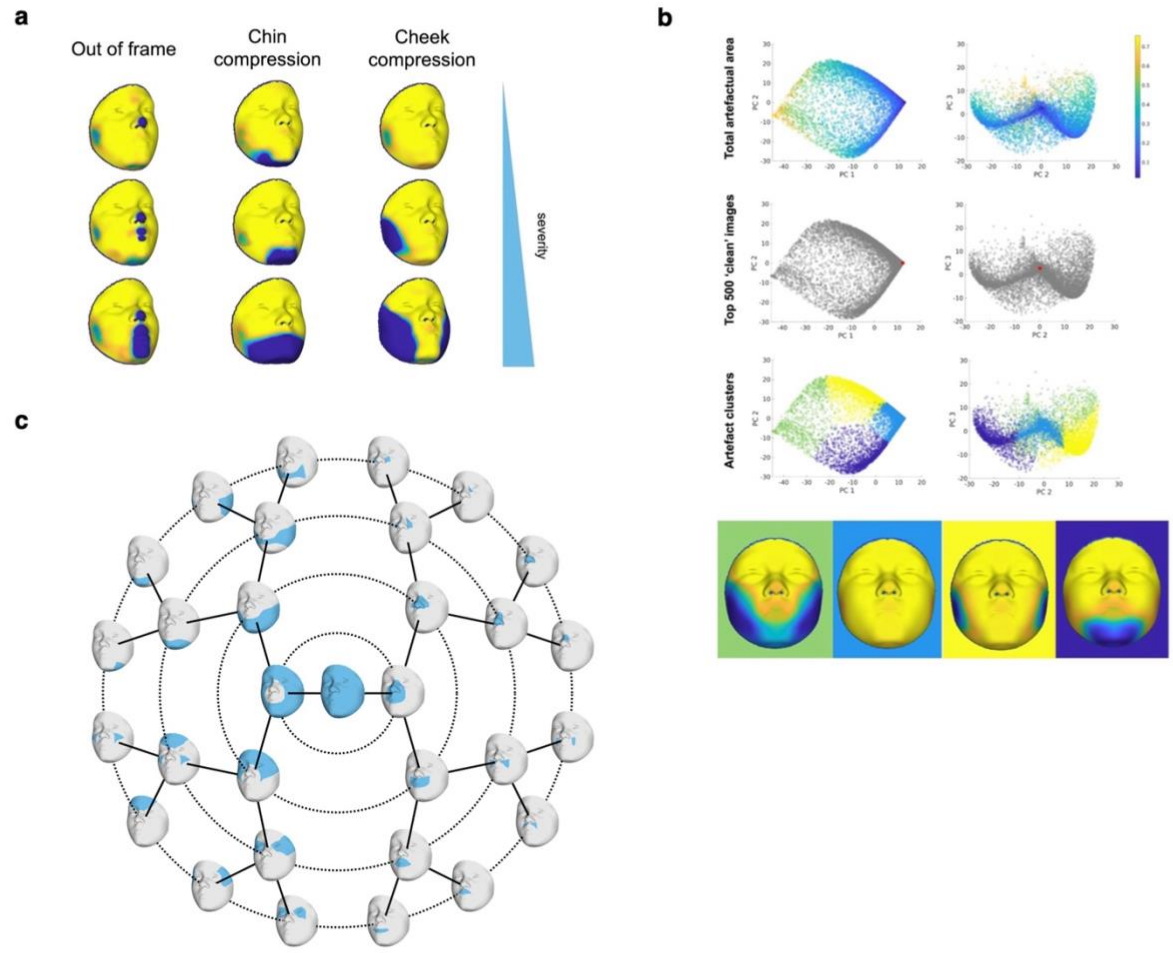

Supplementary Fig. 2. Facial quality control pipeline after surface extraction from MRI. (a) Outlier maps resulting from vertex-wise artefact detection. Different types of artefacts with varying severity could be succesfully be detected following vertex-wise artefact detection. Towards blue indicates high outlier probabilities. Each face represents the average of 10 faces with similar artefacts. **(b)** Detection of artefact clusters using K-means++ clustering. Scatter plots show the first and second PC derived from the vertex-wise outlier maps. From top to bottom: proportion of vertices with outlier probability > 0.05; the top 500 (red) images based on the lowest artefactual area; the clusters from K-meas++ clustering; mean outlier probabilities for each cluster. Clusters capture distict artefact types. Images in the cyan cluster are considered 'clean'. **(c)** Hierarchical spectral segmentation of facial shape. The dendrogram indicates the hierarchical relation between each facial segment (cyan) and the concentric circles indicate the hierarchical levels.

Supplementary Table 1. Overview of GWAS summary data

Trait	Sample size	PubMed ID	Available from
**Conditional GWAS analysis**			
Cranial vault shape	6772	https://pubmed.ncbi.nlm.nih.gov/37973980/	https://www.ebi.ac.uk/gwas/studies/GCST90270327
Facial shape	8246	https://pubmed.ncbi.nlm.nih.gov/33288918/	https://www.ebi.ac.uk/gwas/studies/GCST90007181
Brain shape	19,644	https://pubmed.ncbi.nlm.nih.gov/33821002/	https://www.ebi.ac.uk/gwas/studies/GCST90012880
Heel bone mineral density	426,824	https://pubmed.ncbi.nlm.nih.gov/30598549/	https://www.ebi.ac.uk/gwas/studies/GCST006979
Height	2,200,007	https://pubmed.ncbi.nlm.nih.gov/36224396/	https://www.ebi.ac.uk/gwas/studies/GCST90245990
Systolic blood pressure	385,798	https://pubmed.ncbi.nlm.nih.gov/34017140/	https://www.ebi.ac.uk/gwas/studies/GCST90014018
Diastolic blood pressure	385,801	https://pubmed.ncbi.nlm.nih.gov/34017140/	https://www.ebi.ac.uk/gwas/studies/GCST90014017
Chronic kidney disease	64,164 cases + 561,055 controls	https://pubmed.ncbi.nlm.nih.gov/31152163/	https://www.ebi.ac.uk/gwas/studies/GCST008064
Platelet count	408,112	https://pubmed.ncbi.nlm.nih.gov/32888494/	https://www.ebi.ac.uk/gwas/studies/GCST90002402
LDL cholesterol	361,194	https://pubmed.ncbi.nlm.nih.gov/36376304/	https://www.ebi.ac.uk/gwas/studies/GCST90179148
Inflammatory bowel disease	25,042 cases + 34,915 controls	https://pubmed.ncbi.nlm.nih.gov/28067908/	https://www.ebi.ac.uk/gwas/studies/GCST004131
Alzheimer's disease	71,880 cases + 383,378 controls	https://pubmed.ncbi.nlm.nih.gov/30617256/	https://ctg.cncr.nl/documents/p1651/AD_sumstats_Jansenetal.txt.gz
Schizophrenia	36,989 cases + 113,075 controls	https://pubmed.ncbi.nlm.nih.gov/25056061/	https://figshare.com/ndownloader/files/28570554
**Genomic and morphological correlations**			
Facial shape (63 segments)	8246	https://pubmed.ncbi.nlm.nih.gov/33288918/	https://www.ebi.ac.uk/gwas/publications/33288918
Brain shape (285 segments)	19,644	https://pubmed.ncbi.nlm.nih.gov/33821002/	https://www.ebi.ac.uk/gwas/publications/33821002
**LocusZoom**			
Cranial vault shape	6772	https://pubmed.ncbi.nlm.nih.gov/37973980/	https://www.ebi.ac.uk/gwas/studies/GCST90270327
Facial shape	8246	https://pubmed.ncbi.nlm.nih.gov/33288918/	https://www.ebi.ac.uk/gwas/studies/GCST90007203
Brain shape	19,644	https://pubmed.ncbi.nlm.nih.gov/33821002/	https://www.ebi.ac.uk/gwas/studies/GCST90012880
**S-LDSC**			
Cranial vault shape	4198	https://pubmed.ncbi.nlm.nih.gov/37973980/	https://figshare.com/ndownloader/files/42480399

References

1. Goovaerts, S. *et al.* Joint multi-ancestry and admixed GWAS reveals the complex genetics behind human cranial vault shape. *Nat. Commun.* **14**, 7436 (2023).
2. Auton, A. *et al.* A global reference for human genetic variation. *Nature* **526**, 68–74 (2015).
3. Bergström, A. *et al.* Insights into human genetic variation and population history from 929 diverse genomes. *Science* **367**, eaay5012 (2020).
4. Alexander, D. H., Novembre, J. & Lange, K. Fast model-based estimation of ancestry in unrelated individuals. *Genome Res.* **19**, 1655–1664 (2009).
5. Purcell, S. *et al.* PLINK: A Tool Set for Whole-Genome Association and Population-Based Linkage Analyses. *Am. J. Hum. Genet.* **81**, 559–575 (2007).
6. Danecek, P. *et al.* Twelve years of SAMtools and BCFtools. *GigaScience* **10**, giab008 (2021).
7. Delaneau, O., Zagury, J.-F., Robinson, M. R., Marchini, J. L. & Dermitzakis, E. T. Accurate, scalable and integrative haplotype estimation. *Nat. Commun.* **10**, 5436 (2019).
8. Herrick, N. & Walsh, S. ILIAD: A suite of automated Snakemake workflows for processing genomic data for downstream applications. 2023.10.11.561910 Preprint at https://doi.org/10.1101/2023.10.11.561910 (2023).
9. Rubinacci, S., Delaneau, O. & Marchini, J. Genotype imputation using the Positional Burrows Wheeler Transform. *PLOS Genet.* **16**, e1009049 (2020).
10. Hagler, D. J. *et al.* Image processing and analysis methods for the Adolescent Brain Cognitive Development Study. *NeuroImage* **202**, 116091 (2019).
11. Casey, B. J. *et al.* The Adolescent Brain Cognitive Development (ABCD) study: Imaging acquisition across 21 sites. *Dev. Cogn. Neurosci.* **32**, 43–54 (2018).
12. Klein, S., Staring, M., Murphy, K., Viergever, M. A. & Pluim, J. P. W. elastix: A Toolbox for Intensity-Based Medical Image Registration. *IEEE Trans. Med. Imaging* **29**, 196–205 (2010).
13. Shamonin, D. P. *et al.* Fast parallel image registration on CPU and GPU for diagnostic classification of Alzheimer’s disease. *Front. Neuroinformatics* **7**, 50 (2014).
14. White, J. D. *et al.* MeshMonk: Open-source large-scale intensive 3D phenotyping. *Sci. Rep.* **9**, 6085 (2019).
15. White, J. D. *et al.* Insights into the genetic architecture of the human face. *Nat. Genet.* **53**, 45–53 (2021).

16. Claes, P. *et al.* Genome-wide mapping of global-to-local genetic effects on human facial shape. *Nat. Genet.* **50**, 414–423 (2018).

17. Robert, P. & Escoufier, Y. A Unifying Tool for Linear Multivariate Statistical Methods: The RV-Coefficient. *J. R. Stat. Soc. Ser. C Appl. Stat.* **25**, 257–265 (1976).

18. Naqvi, S. *et al.* Shared heritability of human face and brain shape. *Nat. Genet.* **53**, 830–839 (2021).

19. Dale, A. M., Fischl, B. & Sereno, M. I. Cortical Surface-Based Analysis: I. Segmentation and Surface Reconstruction. *NeuroImage* **9**, 179–194 (1999).

20. Dickie, E. W. *et al.* Ciftify: A framework for surface-based analysis of legacy MR acquisitions. *NeuroImage* **197**, 818–826 (2019).
