# Supplementary material for: Enhanced insights into the genetic architecture of 3D cranial vault shape using pleiotropy-informed GWAS
Source: Commun Biol. 2025 Mar 15;8:439. doi: 10.1038/s42003-025-07875-6 (PMC11909261; doi:10.1038/s42003-025-07875-6)
Supplement: Supplementary file 2 — Description of Additional Supplementary Files [file 42003_2025_7875_MOESM2_ESM.docx]

Description of Additional Supplementary Files

**File name:** Supplementary Data 1

**Description:** Overview of genomic loci associated with cranial vault shape after merging the cFDR-GWASs leveraging bone mineral density (bmd), facial shape (face), or brain shape (brain).

**File name:** Supplementary Data 2

**Description:** Enrichment of GO Biological processes in GREAT, based on the set of merged cFDR-GWAS loci significant at 1% cFDR.

**File name:** Supplementary Data 3

**Description:** Enrichment of GO Biological processes in GREAT, based on the set of merged cFDR-GWAS loci significant at 5% cFDR.

**File name:** Supplementary Data 4

**Description:** Enrichment of Mouse Phenotypes in GREAT, based on the set of merged cFDR-GWAS loci significant at 1% cFDR.

**File name:** Supplementary Data 5

**Description:** Enrichment of Mouse Phenotypes in GREAT, based on the set of merged cFDR-GWAS loci significant at 5% cFDR.

**File name:** Supplementary Data 6

**Description:** Cross-trait polygenic enrichment with cranial vault shape at TF binding sites obtained from TFlink.

**File name:** Supplementary Data 7

**Description:** Output of REVIGO analysis.

**File name:** Supplementary Data 8

**Description:** Source data for manuscript figures.
