# Supplementary material for: Enhanced insights into the genetic architecture of 3D cranial vault shape using pleiotropy-informed GWAS
Source: Commun Biol. 2025 Mar 15;8:439. doi: 10.1038/s42003-025-07875-6 (PMC11909261; doi:10.1038/s42003-025-07875-6)
Supplement: Supplementary file 5 — Reporting Summary [file 42003_2025_7875_MOESM5_ESM.pdf]

nature portfolio

Corresponding author(s): Seppe Goovaerts

Last updated by author(s): Jun 19, 2025

Reporting Summary

Nature Portfolio wishes to improve the reproducibility of the work that we publish. This form provides structure for consistency and transparency in reporting. For further information on Nature Portfolio policies, see our Editorial Policies and the Editorial Policy Checklist.

Statistics

For all statistical analyses, confirm that the following items are present in the figure legend, table legend, main text, or Methods section.

n/a	Confirmed	
☐	☒	The exact sample size (*n*) for each experimental group/condition, given as a discrete number and unit of measurement
☐	☒	A statement on whether measurements were taken from distinct samples or whether the same sample was measured repeatedly
☐	☒	The statistical test(s) used AND whether they are one- or two-sided<br>*Only common tests should be described solely by name; describe more complex techniques in the Methods section.*
☐	☒	A description of all covariates tested
☐	☒	A description of any assumptions or corrections, such as tests of normality and adjustment for multiple comparisons
☐	☒	A full description of the statistical parameters including central tendency (e.g. means) or other basic estimates (e.g. regression coefficient) AND variation (e.g. standard deviation) or associated estimates of uncertainty (e.g. confidence intervals)
☐	☒	For null hypothesis testing, the test statistic (e.g. *F*, *t*, *r*) with confidence intervals, effect sizes, degrees of freedom and *P* value noted<br>*Give P values as exact values whenever suitable.*
☒	☐	For Bayesian analysis, information on the choice of priors and Markov chain Monte Carlo settings
☒	☐	For hierarchical and complex designs, identification of the appropriate level for tests and full reporting of outcomes
☐	☒	Estimates of effect sizes (e.g. Cohen's *d*, Pearson's *r*), indicating how they were calculated

Our web collection on statistics for biologists contains articles on many of the points above.

Software and code

Policy information about availability of computer code

Data collection	No software was used for data collection as part of this study
Data analysis	KU Leuven provides the MeshMonk v.0.0.6 spatially dense facial-mapping software, free to use for academic purposes available at (https://github.com/TheWebMonks/meshmonk). The latest version is available from the FigShare repository of a previous publication (https://doi.org/10.6084/m9.figshare.c.6858271.v1). Matlab implementations of the hierarchical spectral clustering to obtain facial segmentations are available from a previous publication (https://doi.org/10.6084/m9.figshare.7649024.v1). The conditional FDR software is available Github (https://github.com/precimed/pleiofdr).<br><br>The statistical analyses in this work were based on functions in Matlab 2021a–2023b, python v3.7.6, R v4.2.1, PLINK 2.0, bcftools v1.10.2, vcftools v0.1.17, SHAPEIT v4.2.2, IMPUTE5 v1.1.5, imp5Chunker v1.1.5, ADMIXTURE v1.3.0, MeshMonk v0.0.6, GREAT v4.0.4, FUMA v1.6.1, SimpleITK v 2.1.0, REVIGO v1.8.1, FreeSurfer v6.0.0, StringDB v12.0, LDSC v1.0.1

For manuscripts utilizing custom algorithms or software that are central to the research but not yet described in published literature, software must be made available to editors and reviewers. We strongly encourage code deposition in a community repository (e.g. GitHub). See the Nature Portfolio guidelines for submitting code & software for further information.

Data

Policy information about availability of data

All manuscripts must include a data availability statement. This statement should provide the following information, where applicable:

- Accession codes, unique identifiers, or web links for publicly available datasets
- A description of any restrictions on data availability
- For clinical datasets or third party data, please ensure that the statement adheres to our policy

All the data and detailed information for the ABCD Study, including MRI scans, genetic markers, and covariates are available under restricted access through the ABCD data repository (https://nda.nih.gov/abcd/) upon completion of the relevant data use agreements. The ABCD data repository grows and changes over time and the data used in this work came from data release 3.0 (https://doi.org/10.15154/1519007 and https://doi.org/10.15154/wthp-7h18). The NYGC 30×1000 genomes phased dataset and HGDP dataset are freely available online (http://ftp.1000genomes.ebi.ac.uk/vol1/ftp/data_collections/1000G_2504_high_coverage/working/20201028_3202_phased/, and https://ftp.sra.ebi.ac.uk/1000g/ftp/data_collections/HGDP/data/). The LD block coordinates used in this study are available from Berisa et al.137 at (https://bitbucket.org/nygcresearch/ldetect-data/src/master/). Mesh templates used for surface registration are available from the FigShare repositories of previous works (https://doi.org/10.6084/m9.figshare.c.6858271.v1145, https://doi.org/10.6084/m9.figshare.7649024.v1146, and https://doi.org/10.6084/m9.figshare.c.5089841.v1147). Summary statistics for the cFDR-GWASs are available from FigShare (https://doi.org/10.6084/m9.figshare.c.7680035.v1148). An overview of PubMed IDs and URLs for the GWAS summary statistics used in this work are provided in Supplementary Table 1. Transcription factor binding site coordinates can be obtained from the TF Link database v1.0 (https://tflink.net/download/, and https://cdn.netbiol.org/tflink/download_files/TFLink_Homo_sapiens_bindingSites_All_annotation_v1.0.tsv.gz). A list of genomic regions differentially accessible upon TWIST1 loss or depletion can be obtained from Kim et al.83 (GEO: GSE230319; https://www.ncbi.nlm.nih.gov/geo/query/acc.cgi?acc=GSE230319). RefSeq gene and exon annotations used in LocusZoom plots are freely available from the UCSC golden path (http://hgdownload.soe.ucsc.edu/goldenPath/hg19/bigZips/genes/hg19.ncbiRefSeq.gtf.gz). The Source data behind the graphs in the paper can be found in Supplementary Data 1–7 and Source Data.

Research involving human participants, their data, or biological material

Policy information about studies with human participants or human data. See also policy information about sex, gender (identity/presentation), and sexual orientation and race, ethnicity and racism.

Reporting on sex and gender	The current work focuses on aspects of human morphological variation that are independent of sex. Therefore, we include sex assigned at birth in our statistical models as a covariate. No sex-stratified analyses were performed.
Reporting on race, ethnicity, or other socially relevant groupings	No social labels were used to refer to groups of people, rather we have used genetically determined ancestry labels to describe their recent ancestry.
Population characteristics	The ABCD baseline data release (3.0) contains full head high-resolution MRI images for 11,878 children, age 9-10. Participants were genotyped using the Affymetrix NIDA SmokeScreen Array at 733,293 markers. Data collection was done at 21 sites across the US and ABCD adopted epidemiologically informed procedures to ensure that the demographic variation in its sample would mirror the variation in the US population of 9- and 10-year-olds.
Recruitment	We analyzed population cohort data for which participants were recruited in previous studies.
Ethics oversight	Data available through the NIMH data archive has been approved for broad sharing and local institutional approval (S60568) was granted for access to this datasets.

Note that full information on the approval of the study protocol must also be provided in the manuscript.

Field-specific reporting

Please select the one below that is the best fit for your research. If you are not sure, read the appropriate sections before making your selection.

☒ Life sciences ☐ Behavioural & social sciences ☐ Ecological, evolutionary & environmental sciences

For a reference copy of the document with all sections, see nature.com/documents/nr-reporting-summary-flat.pdf

Life sciences study design

All studies must disclose on these points even when the disclosure is negative.

Sample size	No statistical method was used to predetermine sample size for any analysis performed in this work. The main analyses of the work focus on summary level data, i.e., GWAS summary statistics, which were obtained from public repositories. We selected recent GWAS studies with large sample sizes (large in comparison with other GWAS studies on the same trait).<br><br>For the analysis on MRI-derived 3D shape, sample size was maximized based on data availability in the ABCD data repository after excluding samples that failed image processing or were outliers with respect to covariates.
Data exclusions	GWAS studies, other than the cranial vault shape GWAS, involving the ABCD study were automatically excluded due to sample overlap.

	MRI scans that failed QC at any point in the pipeline were excluded as described in the Methods, as well as participants with extreme or missing covariate values or participant who did not cluster with the main (European) inferred recent ancestry cluster. These measures were determined prior to performing any analysis. Exclusions based on ancestry were motivated by the prevalence of imaging artefacts that differentially affected individuals of different recent ancestry, such as braided hair, which could potentially confound results.
Replication	The limited availability of linked craniofacial and genetic data poses significant challenges for both genomic discovery and replication efforts, especially for the cranial vault. While 3D facial data can be collected through 3D surface scanning, this is much more difficult to achieve for the cranial vault due to the presence of hair. Due to the low power of previous cranial vault shape GWAS, only a few of our loci were previously identified.
Randomization	Image-derived shapes from the ABCD study were adjusted for sex, age, height, weight, size, and 10 principal components representing global ancestry components.
Blinding	Blinding was not relevant to our study as we did not compare cases and controls. Investigators did not have access to identifying information.

Reporting for specific materials, systems and methods

We require information from authors about some types of materials, experimental systems and methods used in many studies. Here, indicate whether each material, system or method listed is relevant to your study. If you are not sure if a list item applies to your research, read the appropriate section before selecting a response.

Materials & experimental systems

n/a	Involved in the study
☒	☐ Antibodies
☒	☐ Eukaryotic cell lines
☒	☐ Palaeontology and archaeology
☒	☐ Animals and other organisms
☒	☐ Clinical data
☒	☐ Dual use research of concern
☒	☐ Plants

Methods

n/a	Involved in the study
☒	☐ ChIP-seq
☒	☐ Flow cytometry
☐	☒ MRI-based neuroimaging

Plants

Seed stocks	*Report on the source of all seed stocks or other plant material used. If applicable, state the seed stock centre and catalogue number. If plant specimens were collected from the field, describe the collection location, date and sampling procedures.*
Novel plant genotypes	*Describe the methods by which all novel plant genotypes were produced. This includes those generated by transgenic approaches, gene editing, chemical/radiation-based mutagenesis and hybridization. For transgenic lines, describe the transformation method, the number of independent lines analyzed and the generation upon which experiments were performed. For gene-edited lines, describe the editor used, the endogenous sequence targeted for editing, the targeting guide RNA sequence (if applicable) and how the editor was applied.*
Authentication	*Describe any authentication procedures for each seed stock used or novel genotype generated. Describe any experiments used to assess the effect of a mutation and, where applicable, how potential secondary effects (e.g. second site T-DNA insertions, mosiacism, off-target gene editing) were examined.*

Magnetic resonance imaging

Experimental design

Design type	Resting state
Design specifications	All design specifications can be found in: https://abcdstudy.org/wp-content/uploads/2019/12/Brochure_Protocol-Baseline-eg.pdf
Behavioral performance measures	Not applicable

Acquisition

Imaging type(s): T1 structural imaging

Field strength: 3 Tesla

Sequence & imaging parameters: All parameters for the different MRI scanners can be found in https://abcdstudy.org/images/Protocol_Imaging_Sequences.pdf

Area of acquisition: Whole brain/head scan

Diffusion MRI: ☐ Used ☒ Not used

Preprocessing

Preprocessing software:

Minimally processed T1 structural MRI data were downloaded from the ABCD data release 3.0. The processing pipeline is available from ABCD as a docker container from https://github.com/ABCD-STUDY/abcd_docker. Used software include: Freesurfer (v7.1.1), FSL (v5.0.2.2-centos6_64), AFNI (v2010_10_19_1028), MMP (v2.5.1), Dcm2niix, dtitk (v2.3.1-Linux-x86_64), gosu (v1.11), Matlab Compiler Runtime (v8.4), dcmtk (v3.6.0). See Hagler et al., 2019 (10.1016/j.neuroimage.2019.116091) for a detailed description of MRI preprocessing methodology and software.

We used the Elastix toolbox (SimpleITK v 2.1.0) to remove noise from the outer head surface; and Meshmonk (v0.0.6) to perform non-rigid surface registration using a full head or facial surface template as described in the Methods.

Freesurfer (v6.0.0) was used to extract and process mid-cortical brain shape from the MRIs , using a template from the Human Connectome Project.

Normalization:

First, MRI images were corrected for gradient nonlinearity distortions using scanner-specific, nonlinear transformations provided by MRI scanner manufacturers. Second, ABCD performed bias field correction using a novel implementation that is similar in purpose to commonly used bias field correction methods. Finally, images were resampled to 1.0 mm isotropic voxels. See Hagler et al., 2019 (10.1016/j.neuroimage.2019.116091) for a detailed description of MRI preprocessing methodology and software.

Non-rigid surface registration was performed on MRI scans after artifact denoising (see below) using a full-head surface template, and the cranial vault surface comprising 11,410 vertices was subsequently selected.

Cranial vault, face, and brain configurations were adjusted for covariates (sex at birth, age, weight, height, cranial size, and 10 genomic ancestry PCs) using partial least squares regression in Matlab 2023a.

Normalization template:

For normalization of MRI images, ABCD used a standard reference brain with 1.0 mm isotropic voxels obtained from averaging 500 adult brain nonlinearly registered to an initial template.

For non-rigid surface registration, a cranial vault template was obtained from Goovaerts et al. (2023); a facial template (n = 7,160 vertices) was obtained from White et al. (2021); and a cortical brain template was obtained from the Human Connectome Project.

Noise and artifact removal:

We generated virtual re-acquisitions by an inter-subject intra-MRI non-rigid image-based registration approach. A total of 300 MRI scans ('floating' scans) – matched in terms of sex at birth, height, weight, and genomic ancestry – were registered to a single target MRI scan using Elastix (SimpleITK library in Python) with the Param0000 parameter map (affine and B-spline). The use of 300 floating scans per 'target' image was chosen based on visual inspection of the results while controlling for computational time and resources. The resulting, denoised consensus 'target' image was defined as the voxel-per-voxel median of the resulting warped 'floating' images.

Volume censoring: Not applicable

Statistical modeling & inference

Model type and settings: Multivariate shape analysis

Effect(s) tested: Cranial vault shape variation explained by the face and brain.

Specify type of analysis: ☐ Whole brain ☒ ROI-based ☐ Both

Anatomical location(s): Anatomical regions were defined based on the templates available from previous studies. Brain shape is defined as the mid-cortical surface; the cranial vault as the outer head surface extending from the orbitals towards the occipital bone; and facial shape as the facial soft-tissue surface including the chin and forehead.

Statistic type for inference (See Eklund et al. 2016): The surface-to-surface variance explained was estimated using partial least squares regression.

Correction | Correction of multivariate shape variables for covariates was performed using partial least squares regression.

Models & analysis

n/a	Involved in the study
☒	☐ Functional and/or effective connectivity
☒	☐ Graph analysis
☐	☒ Multivariate modeling or predictive analysis

Multivariate modeling and predictive analysis

For the face, brain, and cranial vault separately, the set of 3D surface vertices in the segment were subjected to a GPA. A shape-space for each trait was built by conducting PCA on the pooled x, y, and z coordinates of each vertex within the segment and parallel analysis was subsequently used to retain the major axes of shape variation. Partial least squares regression was performed to estimate the proportion of cranial vault shape variation explained by the face or brain.
